# Sex specific molecular networks and key drivers of Alzheimer’s disease

**DOI:** 10.1186/s13024-023-00624-5

**Published:** 2023-06-20

**Authors:** Lei Guo, Jiqing Cao, Jianwei Hou, Yonghe Li, Min Huang, Li Zhu, Larry Zhang, Yeji Lee, Mariana Lemos Duarte, Xianxiao Zhou, Minghui Wang, Chia-Chen Liu, Yuka Martens, Michael Chao, Alison Goate, Guojun Bu, Vahram Haroutunian, Dongming Cai, Bin Zhang

**Affiliations:** 1 Department of Genetics and Genomic Sciences, Icahn School of Medicine at Mount Sinai, New York, NY 10029 USA; 2 Mount Sinai Center for Transformative Disease Modeling, Icahn School of Medicine at Mount Sinai, New York, NY 10029 USA; 3 Department of Neurology, Icahn School of Medicine at Mount Sinai, New York, NY 10029 USA; 4 James J Peters VA Medical Center, Research & Development, Bronx, NY 10468 USA; 5 Department of Neuroscience, Mayo Clinic, Jacksonville, FL 32224 USA; 6 Department of Neuroscience, Yale University, New Haven, CT 06510 USA; 7 Ronald M. Loeb Center for Alzheimer’s Disease, Icahn School of Medicine at Mount Sinai, New York, NY 10029 USA; 8 Alzheimer Disease Research Center Icahn School of Medicine at Mount Sinai, New York, NY 10029 USA; 9 Department of Psychiatry, Icahn School of Medicine at Mount Sinai, New York, NY 10029 USA; 10 James J Peters VA Medical Center, MIRECC, Bronx, NY 10468 USA; 11 Department of Pharmacological Sciences, Icahn School of Medicine at Mount Sinai, New York, NY 10029 USA

**Keywords:** Alzheimer’s disease, Sex difference, Gene co-expression network, Key driver genes, APOE genotype, LDL receptor related protein 10 (LRP10)

## Abstract

**Background:**

Alzheimer’s disease (AD) is a progressive and age-associated neurodegenerative disorder that affects women disproportionally. However, the underlying mechanisms are poorly characterized. Moreover, while the interplay between sex and ApoE genotype in AD has been investigated, multi-omics studies to understand this interaction are limited. Therefore, we applied systems biology approaches to investigate sex-specific molecular networks of AD.

**Methods:**

We integrated large-scale human postmortem brain transcriptomic data of AD from two cohorts (MSBB and ROSMAP) *via* multiscale network analysis and identified key drivers with sexually dimorphic expression patterns and/or different responses to APOE genotypes between sexes. The expression patterns and functional relevance of the top sex-specific network driver of AD were further investigated using postmortem human brain samples and gene perturbation experiments in AD mouse models.

**Results:**

Gene expression changes in AD *versus* control were identified for each sex. Gene co-expression networks were constructed for each sex to identify AD-associated co-expressed gene modules shared by males and females or specific to each sex. Key network regulators were further identified as potential drivers of sex differences in AD development. *LRP10* was identified as a top driver of the sex differences in AD pathogenesis and manifestation. Changes of LRP10 expression at the mRNA and protein levels were further validated in human AD brain samples. Gene perturbation experiments in EFAD mouse models demonstrated that *LRP10* differentially affected cognitive function and AD pathology in sex- and APOE genotype-specific manners. A comprehensive mapping of brain cells in *LRP10* over-expressed (OE) female E4FAD mice suggested neurons and microglia as the most affected cell populations. The female-specific targets of *LRP10* identified from the single cell RNA-sequencing (scRNA-seq) data of the *LRP10* OE E4FAD mouse brains were significantly enriched in the *LRP10*-centered subnetworks in female AD subjects, validating *LRP10* as a key network regulator of AD in females. Eight LRP10 binding partners were identified by the yeast two-hybrid system screening, and LRP10 over-expression reduced the association of LRP10 with one binding partner CD34.

**Conclusions:**

These findings provide insights into key mechanisms mediating sex differences in AD pathogenesis and will facilitate the development of sex- and APOE genotype-specific therapies for AD.

**Supplementary Information:**

The online version contains supplementary material available at 10.1186/s13024-023-00624-5.

## Background

Alzheimer’s disease (AD) is a progressive and age-associated neurodegenerative disorder. It affects women disproportionally as manifested in many aspects such as disease prevalence, clinical presentation, neuroimaging studies and treatment responsiveness from clinical trials [1]. Of all AD patients in the United States, around two-thirds are women [2]. The lifetime risk for developing AD is two times higher in women than men [3]. Women with AD show greater cognitive vulnerability to AD pathology, steeper rates of cognitive decline, and faster brain volume loss than men do [4, 5]. The brain atrophy rate is 1–1.5% faster in women with AD than that in men [6]. The association of AD pathology with clinical manifestations of the disease is also more significant in women than men [4]. At molecular levels, the link between AD and a genetic risk factor, *APOE4* is much more prominent in women than men [7, 8]. Therefore, the growing body of evidence supporting sex differences in AD highlights the importance of understanding the molecular architecture of underlying female and male AD brains. Despite the continuous effort in the field, the molecular and cellular mechanisms underlying sex differences in AD pathogenesis remain poorly understood [9].

In this study, we investigated sex-specific molecular networks of AD using unbiased systems biology approaches to analyze the transcriptomic data of 338 postmortem human brain samples from the Mount Sinai Brain Bank (MSBB) cohort and the Religious Orders Study and Rush Memory and Aging Project (ROSMAP) cohort. After quality control and covariate correction of the assembled data to ensure that sex status was correctly annotated and that covariates such as age did not confound our analyses, we identified genes differentially expressed between females and males, as well as between APOE4 carriers and non-carriers in AD. We then performed a multiscale co-expression network analysis of sex specific transcriptomic data to identify key subnetworks and regulators responsible for sex differences in AD development. Among the candidate genes, lipoprotein receptor related protein 10 (*LRP10*) was identified as a top key regulator of female AD network that potentially drives sex differences in AD development based on its high regulatory strength and network connectivity, sex-specific differential expression significance as well as APOE4 dosage dependency in AD. We further validated the changes of LRP10 at the gene and protein expression levels using an independent cohort of postmortem human brain samples from the para-hippocampal gyrus (PHG), a brain region mostly associated with AD pathology [10] to confirm the biological relevance of the findings. The subsequent gene perturbation experiments in EFAD mouse models further confirmed that *LRP10* as a key network regulator of AD in females affected cognitive function and AD-related pathology in sex- and APOE genotype-specific manners. The downstream signaling pathways of LRP10 were further characterized by the comprehensive brain cell type mapping through the scRNA-seq analysis as well as identification of LRP10 binding partners by the yeast two-hybrid system studies.

## Methods

### RNA-seq gene expression profile and data preprocessing

Gene expression data were generated from two different brain regions: the para-hippocampal gyrus (PHG) from the Mount Sinai/JJ Peters VA Medical Center Brain Bank (MSBB) AD cohort [10] and the prefrontal cortex from the ROSMAP cohort [11]. The MSBB raw sequencing reads were aligned to the human hg19 genome (Star Aligner version 2.5.0b) and quantified using featureCounts [12] based on the Ensembl gene model GRCh37.70. Genes with at least 1 count per million (CPM) in at least one sample were selected, normalized [13] and corrected for covariates such as postmortem interval (PMI), race, RNA integrity number (RIN), rate of exonic reads, and batch using a linear mixed model. The preprocessed RNA-seq FPKM gene expression abundance data of the ROSMAP cohort was obtained (Synapse doi:10.7303/syn3388564), and genes with at least 1 FPKM in at least 10% of the samples were selected and the data was corrected for covariates including batch, PMI and RIN. A total of 23,201 genes in the MSBB cohort and 16,387 genes in the ROSMAP cohort were interrogated. There are 788 genes on the X chromosome and 42 genes on the Y chromosome in the MSBB data, and 546 genes on the X chromosome and 18 on the Y chromosome in the ROSMAP data.

### Clinical and pathological data

The neuropathological assessments for the Mount Sinai Brain Bank (MSBB) AD cohort samples were performed following procedures previously described in detail [14–17], including Braak stage [18, 19], clinical dementia rating (CDR) scale [20], the Consortium to Establish a Registry for Alzheimer's Disease (CERAD) score [21], and mean plaque density [14]. The clinical traits, cognitive assessments and disease stages of subjects from the MSBB cohort [22, 23] and the ROSMAP cohort [11] have been previously described in detail as well.

### Differential expression analysis and trend analysis

The differential expression (DE) analysis was performed between different disease severity stages, between female and male, and between different APOE subgroups using R package limma (V3.34.0) with default settings [24]. AD was diagnosed by a combination of Braak stage and CERAD score. Samples diagnosed as possible or probable AD were not included. Disease stages were determined by CDR, Braak stage, CERAD or Plaque Density. By Braak stage, we defined normal (stage <  = 2), medium (2 < stage <  = 4) and severe (stage > 4) groups. By CDR, we also defined normal (CDR = 0), MCI (CDR = 0.5) and AD (CDR > 0.5) groups. We further classified the cohort subjects by plaque mean density (PMD) into low (PMD <  = 6), medium (6 < PMD <  = 12) and high (PMD > 12) groups. The information for each human sample for both cohorts (MSBB and ROSMAP) was provided in Supplemental Table 1A-B while Supplemental Table 1C showed the statistical summary of different subgroups. The numbers of differentially expressed genes (DEGs) from the DE analysis of various subgroup comparisons can be found in Supplemental Table 2A. Multiple tests were adjusted using the Benjamini–Hochberg’s (BH) FDR method. Genes with an FDR adjusted *p* value less than 0.05 and fold change (FC) greater than 1.2 were considered significant. To test if the expression pattern of a gene is differentially associated with AD progression or affected by APOE genotype between females and males, the trend analysis on each gene for each clinical trait (Braak stage, CDR, CERAD and plaque density) and APOE genotype (ε23, ε33, ε34) in each sex was performed using the Jonckheere trend analysis method to capture linear trends and the Spline trend analysis method for non-linear trends across these features. Multiple tests were adjusted using the Benjamini–Hochberg’s (BH) FDR method. Genes showing significantly opposite trends between females and males for each clinical trait or APOE genotype were selected for further analysis.

### Gene co-expression network analysis

Multiscale embedded Gene co-Expression Network Analysis (MEGENA) [25] was performed to construct AD- and sex-specific gene regulatory networks for the identification of co-expressed gene modules in each brain region from AD subjects. Spearman’s rank correlation coefficient analysis was used to compute the strength of correlation between AD clinical traits (i.e., CDR, plaque, CERAD, Braak stage) and modules [10]. Multiple tests were adjusted using the Benjamini–Hochberg’s (BH) FDR method [26]. *P* values from the module-trait correlation analyses and those from the module-TCG enrichment analyses were combined (i.e., $\sum_{i}-log10\left({P}_{i}\right)$) to rank-order modules in each gene co-expression network. The module-TCG enrichment analysis was based on the gene signatures correlated with AD clinical traits (i.e., CDR, plaque, CERAD, Braak stage). Following the established procedure of key driver analysis (KDA) [27], we nominated genes with high connectivity (greater than the mean connectivity plus one standard deviation) in a module as key network drivers (KND). The modular differential connectivity (MDC) analysis [28] was carried out to detect and quantify the network reorganization between female and male AD patients. The Gene Ontology (GO) Consortium and Kyoto Encyclopedia of Genes and Genomes (KEGG) databases were applied for functional enrichment and pathway analysis of modules.

### Identification of key sex-specific driver genes of AD

The AD-associated modules from the MSBB and ROSMAP cohorts were used in the identification of sex-specific key driver genes of AD. By definition, a driver gene for AD should regulate a number of other genes related to AD, therefore, a candidate sex-specific driver of AD must be a driver of a module associated with AD in a sex specific gene co-expression network. On top of this, a sex specific candidate driver should show 1) differential expression between AD and control and between females and males in AD, or 2) opposite expression trends between females and males in at least two clinical traits, or 3) opposite expression trends between females and males across *APOE* genotypes. Key network driver genes that meet any of the above three criteria were selected and further rank-ordered by the strength of association with AD. Identification of sex specific key drivers of AD utilized three complementary strategies including differential expression analysis (DEA), trait-based differential trend analysis (TDTA) and *APOE* genotype based differential trend analysis (ADTA). The association of KNDs with AD was assessed by the enrichment of the DEGs (female AD *versus* female control, male AD *versus* male control and female AD *versus* male AD) in the neighbors of the 3-layer neighbors of the sex-specific gene co-expression networks of that specific candidate gene (female AD networks for female candidate genes and male AD network for male candidate genes). The rank order was calculated based on multiple *p* values calculated from module-traits correlation and module-DEG enrichment analyses.

### Validation of LRP10 female-specific expression patterns in human brain samples

The LRP10 mRNA and protein expression levels were determined using post-mortem human brain samples derived from the PHG region of the MSBB cohort with *APOE3/3 versus APOE3/4* genotypes, female *versus* male subjects of normal aging (CDR: 0–0.5) and AD (CDR: 0.5–3) provided by the NIH NeuroBioBank brain and tissue repository (NBTR).

### Animal models

Human *APOE4*^+*/*+^ or *APOE3*^+*/*+^ knock-in (KI) mouse models with 5xFAD background [29, 30] were genotyped as described. All animal experiments were performed by following the NIH guidelines and were approved by the JJPVAMC and ISMMS Institutional Animal Care and Use Committees (IACUC). Sex as a biological variable was taken into considerations with inclusion of both male and female mice in all experiments.

### Stereotaxic injection and behavior studies

8–9 weeks old male and female *APOE3*^+*/*+^ and *APOE4*^+*/*+^ KI mice with 5xFAD background (*N* = 15–18/group) were placed in the stereotaxic apparatus with AAV2/9-containing LRP10 or scramble control virus administered into the dorsal CA1 regions of bilateral hippocampal brain regions using pressure injection as described [31, 32]. Injection volumes (0.5–2.0 µl) were delivered over 10 min to avoid tissue damage. 6–9 months after viral delivery, mice were tested with the NOR task and Y maze as described [33–35]. Mice were randomized for genotype and sex, and blinded throughout the behavior data collection and analysis, surgical manipulations, and sample collection following the NIH practice guidelines. Animals were excluded from behavior analysis if the total exploration time was less than 4 s, the total arm entry was less than 10, or if they had an illness that prevented them from reliably completing the behavior tests.

### Brain and neuronal sample preparation and biochemical analysis

Snap-frozen mouse hemi-brains were harvested in lysis buffer [36] and processed via step-wise solubilization [36, 37], followed by SDS-PAGE to determine levels of LRP10, CD34, NBR1, ACBD3, LRP1, LRP3, LRP6, LDLR, pTau, total tau and β-actin. Levels of Aβ_42_, Aβ_40_, APOE and cytokines (IL6, IL10, IL17 and TNFα) were determined using high-sensitive ELISA kits. Some tissue was used for RNA extraction followed by qPCR and RNA-seq analysis. Some fresh tissue was used for scRNA-seq analysis. Some animals underwent perfusion followed by brain tissue section for immunohistochemical staining of amyloid plaque and IBA1.

### Bulk tissue RNA-seq and single-cell RNA-seq analysis

The RNA was extracted from male and female E3FAD and E4FAD mouse hippocampal brain tissue from LRP10 over-expression or control conditions (*N* = 5/group), followed by generation of RNA-seq libraries with a Hi-Seq 4000 platform. In parallel, the single-cell suspensions were prepared from EFAD mouse brains (hippocampus) with LRP10 over-expression or control (*N* = 4/group), processed with the 10X Genomics Chromium platform. Cells with at least 200 genes expressed, a mitochondrial read rate of less than 20% and a ribosome read rate of more than 5% were considered viable and were kept for the subsequent analyses. Cells with abnormally high UMIs and genes expressed were considered as multiplets and were removed. Genes that were detected in more than 3 cells were kept. After quality control (QC), the gene-level UMIs data were normalized by a regularized negative binomial regression analysis [38]. The principal component analysis (PCA) was performed with significant principal components determined by a JackStraw permutation procedure to select cell clustering using Seurat’s graph-based clustering approaches [39]. The normalized dataset was projected onto the Uniform Manifold Approximation and Projection for Dimension Reduction (UMAP) [40]. The cell type marker genes were interrogated to annotate major cell-type clusters including astrocytes, neurons, oligodendrocytes, microglia, endothelial cells and oligodendrocyte progenitor cell (OPC) [41].

### Differential expression analysis of bulk tissue RNA-seq and scRNA-seq data

Differential gene expression analysis was performed on the LRP10 over-expression group and the control group in each sex group (male and female) in each APOE genotype (E3 and E4) of each cell population (cluster). Significant DEGs (FDR adjusted *p* < 0.05) in each sex group were then combined as a union into a single gene signature considered as the sex-specific targets of *LRP10*.

### Yeast two-hybrid screen and assay

The yeast two-hybrid screen with human LRP10 cytoplasmic tail as a bait was screened against the Human Fetal Brain MATCHMAKER cDNA Library (CLONTECH Laboratories) by Creative Biolabs (Shirley, New York, USA). Briefly, the bait gene coding for the human LRP10 cytoplasmic tail was synthesized and subcloned into the vector pGB. Toxicity effects and self-activation were tested by the β-galactosidase assays, and no toxicity and self-activation were observed from pGB-LRP10tail. The yeast transformed with pGB-LPR10tail plasmid was cultured and transformed with Human Fetal Brain cDNA library for screening. A total of 13 positive clone transformants were picked and validated in filter detection by the β-galactosidase assays. Plasmids from 13 positive clones were extracted and sequenced. Subsequently, the bait and positive clone plasmids were co-transformed back to yeast and detected by the β-galactosidase assays for further verification. Eight unique positive hits were obtained.

### Antibodies and reagents

The anti-LRP10 (rabbit polyclonal Ab, ThermoFisher, RRID:AB_2555821; 1:200), anti-pTau AT8 (mouse monoclonal Ab, ThermoFisher, RRID:AB_223647; 1:1000), anti-Tau-5 (mouse monoclonal Ab, ThermoFisher, RRID:AB_10980631; 1:1000), anti-LRP1 (mouse monoclonal Ab, ABCAM, RRID:AB_445797; 1:500), anti-LRP3 (rabbit monoclonal Ab, ABCAM, RRID:AB_2139009; 1:500), anti-LDLR (rabbit polyclonal Ab, ABCAM, RRID:AB_881272; 1:500), anti-LRP6 (mouse monoclonal Ab, ABCAM, RRID:AB_2139308; 1:500), anti-β-actin (mouse monoclonal Ab, Santa Cruz Biotechnology, RRID:AB_476697; 1:10000), anti-tubulin (mouse monoclonal Ab, Santa Cruz Biotechnology, RRID:AB_477498; 1:5000), anti-beta-Amyloid (Cell Signaling Technology, RRID: AB_2056585; 1:200), anti-IBA1 (mouse monoclonal Ab, ThermoFisher, RRID:AB_2735228; 1:200; and rabbit monoclonal Ab, Abcam; RRID:AB_2636859; 1:200), anti-CD34 (rabbit polyclonal Ab, Abnova, RRID:AB_10891213; 1:500), anti-NBR1 (rabbit polyclonal Ab, ThermoFisher, RRID:AB_2718814; 1:500), anti-ACBD3 (mouse monoclonal Ab, ThermoFisher, RRID:AB_2722803; 1:500), anti-mouse and rabbit HRP (ThermoFisher, RRID:AB_2556542 and 2540618; 1:1000), Texas-Red and Alexa^555^ conjugated anti-mouse and rabbit IgG (ThermoFisher, RRID:AB_10374713, 10983944, 2535987 and 1090271; 1:1000) were purchased. AAV2/9-containing LRP10 and AAV2/9-control vectors were generated and obtained from ABM Inc. The qPCR probes for *actin* (Hs1060665_g1), *lrp10* (Hs01047362_m1 and Mm00499125_m1), and *gapdh* (Mm99999915_g1) were purchased from ThermoFisher. ELISA kits for human amyloid Aβ_40_, Aβ_42_, aggregated oligomer Aβ_42_ and APOE (ThermoFisher), as well as mouse TNFα (ThermoFisher, RRID:AB_2575667), IL-6 (ThermoFisher, RRID:AB_2575651), IL-10 (ThermoFisher, RRID:AB_2575689), IL-17 (ThermoFisher) were also purchased.

### Statistical analysis 

The sample size of each experiment was determined based on power calculations derived from previous similar studies which allowed us to determine group sizes needed to achieve statistically significant results. All experiments including controls were performed in randomly assigned groups. Sample collection and data analysis followed the NIH practice guidelines. Experimenters were blinded to the experimental conditions of the animals while conducting experiments. The conditions were revealed after the quantification was completed. Levels of mRNA of interest were normalized to GAPDH and 18 s, and then expressed as Log_2_fold of changes when compared to controls. Levels of LRP10, pTau, Tau, CD34, NBR1, ACBD3, LRP1, LRP3, LRP6 and LDLR were normalized to β-actin levels and expressed as a percentage of the control. The amounts of CD34, NBR1 and ACBD3 that were associated with LRP10 and pulled down by anti-LRP10 antibody was quantified and expressed as a percentage of the control. Absolute oligomer Aβ_42_, soluble Aβ_42_ and Aβ_40_, IL6, IL10, IL-17, APOE and TNFα concentrations were quantitatively determined by ELISA and expressed as a percentage of the control. Independent-samples *t-*tests were used to determine significant mean differences (the threshold for significance sets at *p* < 0.05). ANOVA with post-hoc tests was used to determine group differences for multiple comparisons. Pearson correlation coefficients were calculated to determine the linear relationship between the two variables. Equality of variance was checked for all statistical comparisons. When independent-sample *t*-tests were used and equality of variances of compared groups were not the same, Welch’s corrections were applied. All statistical analysis was performed using Prism 9.0.

## Results

### Differential gene expression profiles of female and male AD *versus* control

**Fig. 1:**
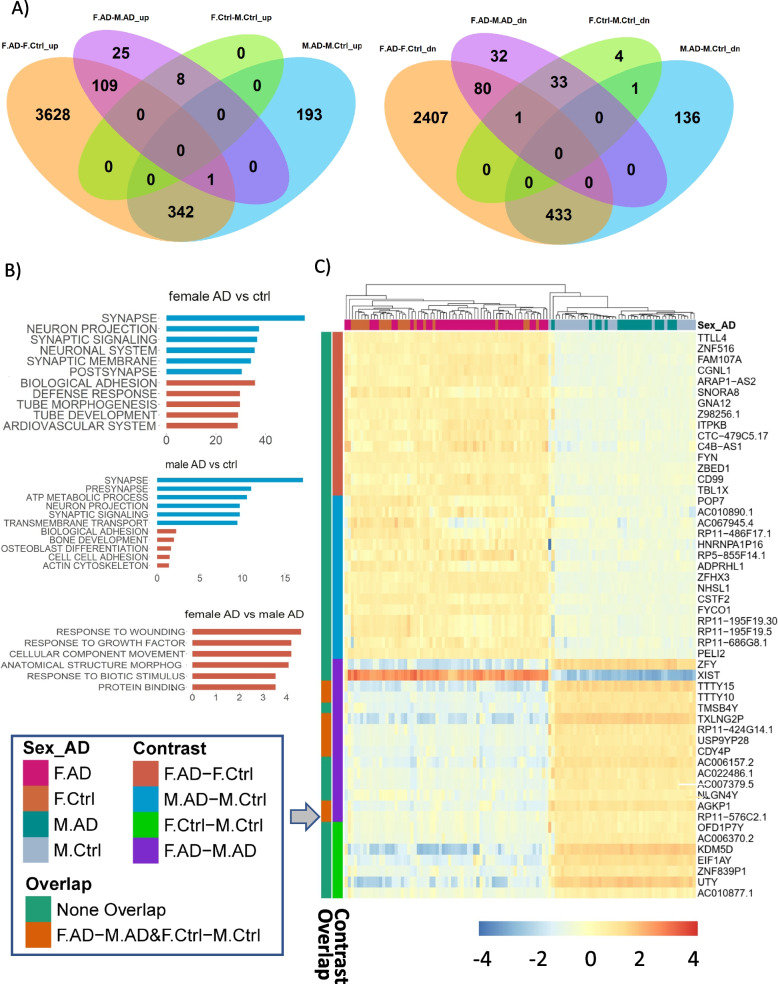
Comparison of Differential Gene Expressed Gene (DEG) Signatures between female AD and female control and between  male AD and male control. **A** Venn diagrams to show the numbers of DEGs identified in different groups of comparison: female AD *versus* female control (F_AD-F_Ctrl), male AD *versus* male control (M_AD-M_Ctrl), female AD *versus* male AD (F_AD-M_AD) and female control *versus* male control (F_Ctrl-M_Ctrl). Left: up-regulated DEGs; Right: down-regulated DEGs. **B** The most enriched functions/pathways for the DEGs identified between AD and the control in women (top) and men (middle), as well as between women and men (bottom). Functions/pathways in blue/red were enriched by the down/up-regulated genes in AD/women; X axes represented –log10 (false discovery rate: FDR). **C** Expression patterns of the top 15 DEGs identified in each of the four comparisons (AD *versus* control in each sex & female *versus* male in each diagnostic category). Colored bars on the left represented the DEGs identified in which comparison and whether there were overlaps among the different comparisons. Color bars on top represented the expression pattern of the genes in the corresponding sex/AD groups

The numbers of differentially expressed genes (DEGs) identified from different comparisons (AD *versus* normal aging control subjects; females *versus* males) were shown in Fig. 1A and Supplemental Fig. 1A. In the PHG region, DEG signatures generated from three comparison groups (female AD *versus* female control, male AD *versus* male control and female AD *versus* male AD) showed significantly enriched GO terms and KEGG pathways. The up-regulated genes in AD females were associated with biological adhesion, defense and immune responses, as well as blood vessel development, while those in males were mostly involved in adhesion and cytoskeleton development (Fig. 1B; Supplemental Table 2). The down-regulated DEGs between female AD *versus* female control as well as male AD *versus* male control were mainly enriched for neuron/synapse functions, whereas the up-regulated DEGs were related to responses to wounding/stimulus and vasculature development (Fig. 1B). Further examination of the top DEGs identified among four comparisons (female AD *versus* female control, male AD *versus* male control, female AD *versus* male AD, and female control *versus* male control) suggested sex-specific DEG signatures (i.e., DEGs between male AD *versus* male control and DEGs between female AD *versus* female control) did not overlap significantly with those between female AD *versus* male AD (Fig. 1C and Supplemental Fig. 1A). However, the DEGs of female AD *versus* male AD significantly overlapped with those between female control *versus* male control, with sex-chromosome linked genes identified as most of the top DEGs (Fig. 1C). The numbers of up- and down-regulated DEGs between female AD *versus* female control were much larger than those between male AD *versus* male control (Fig. 1A). These differences were unlikely due to the under-powered male samples because when we analyzed a down-sampled female dataset, a significantly large number of DEGs between female AD *versus* female control (> 3,000 DEGs) was again observed with the Principal Component Analysis (PCA) showing many more differences between female AD and control than those between male AD and control (Supplemental Fig. 1B).

We further investigated whether sex-specific gene expression changes we observed were biased towards sex-chromosome genes. It was found that the female DEG signatures between AD *versus* control of the PHG (MSBB cohort) and the PFC (ROSMAP cohort) were not enriched for the X-chromosome genes and there was no Y-chromosome gene differentially expressed between AD males *versus* control males in either cohort. In the RNA-seq data from the PHG in the MSBB AD cohort, the female and male DEG signatures shared 38 genes and each of which was differentially expressed in AD in the same direction (up or down). However, in the ROSMAP cohort, the male and female DEG signatures didn’t share any gene. Therefore, our results suggested that the observed sex-specific DEGs in AD were not biased towards sex-chromosome genes in the advanced stage of AD patients studied in the MSBB and the ROSMAP cohorts. However, this doesn’t exclude the possibilities of any potential involvement of sex-chromosome genes in sex-specific gene expression changes in other brain regions or during early or pre-symptomatic stage of AD.

When comparing DEGs between AD *versus* control in each *APOE* and sex subgroup of the PHG data in the MSBB cohort and the PFC data in the ROSMAP, the PHG brain region showed many more significant transcriptional changes between AD *versus* control as well as female *versus* male (Supplemental Figs. 1C and 2A). Furthermore, there were many more male- and female-specific DEGs between AD and control in the PHG than the PFC (Supplemental Figs. 2B). In females, the DEGs shared between the PHG and the PFC account for only 4% of the DEGs of the PHG but 65% of the DEGs in the PFC. In males, the 1,102 DEGs in the PHG and the 32 DEGs in the PFC shared only one gene. Together, these results suggested brain region specific sex-biased gene expression patterns in AD pathogenesis.

The differential expression (DE) analysis on each of the four clinical traits, i.e., clinical dementia rating (CDR), Braak stage, CERAD, and plaque density was also performed on the samples classified into 3 disease stage categories for each trait (normal, low severity and high severity). The resulting DEGs were considered as trait-associated genes (TCGs). To complement the DE analysis, sequential Jonckheere and Spline trend analyses of each gene in females and males across *APOE* genotypes (i.e., *APOE4* dosage) and AD clinical traits were performed to capture both linear and non-linear expression trends (Supplemental Fig. 3A; Supplemental Table 3). For *APOE* genotype based TCGs, 24 with oppositive trends in the female and male groups were considered as potential sex-specific genes for *APOE*: 15 genes with female positive and male negative correlations and 9 genes with male positive and female negative correlations (Supplemental Fig. 3B; Supplemental Table 3). Moreover, genes with a significant trend with respect to each clinical trait and each sex group were determined (Supplemental Fig. 3C and 2D; Supplemental Table 3), and only genes showing opposite trends between females and males were considered as potential sex-specific genes. Genes showing significantly differential expression trends in females and males for at least two clinical traits were identified as differentially trended genes (DTGs). The top three known AD risk genes with significantly opposite trends between female and male for each clinical trait including *APOE*, Braak score, CDR, CERAD, and plaque load were shown (Supplemental Fig. 3E; Supplemental Tables 3). Supplemental Table 3E included the results from the comparison of various DTG signatures in the PHG from the MSBB cohort. As expected, these DTG signatures significantly overlapped with each other (Supplemental Table 3E; Supplemental Fig. 4). While some known AD risk genes were also DTGs, but overall they were not enriched in any DTG signatures** (**Supplemental Table 3F). These results suggested that sex differentially modulates expression patterns of AD risk genes, which could potentially impact sex-biased AD pathogenesis.

### Sex-specific co-expression network modules

**Fig. 2:**
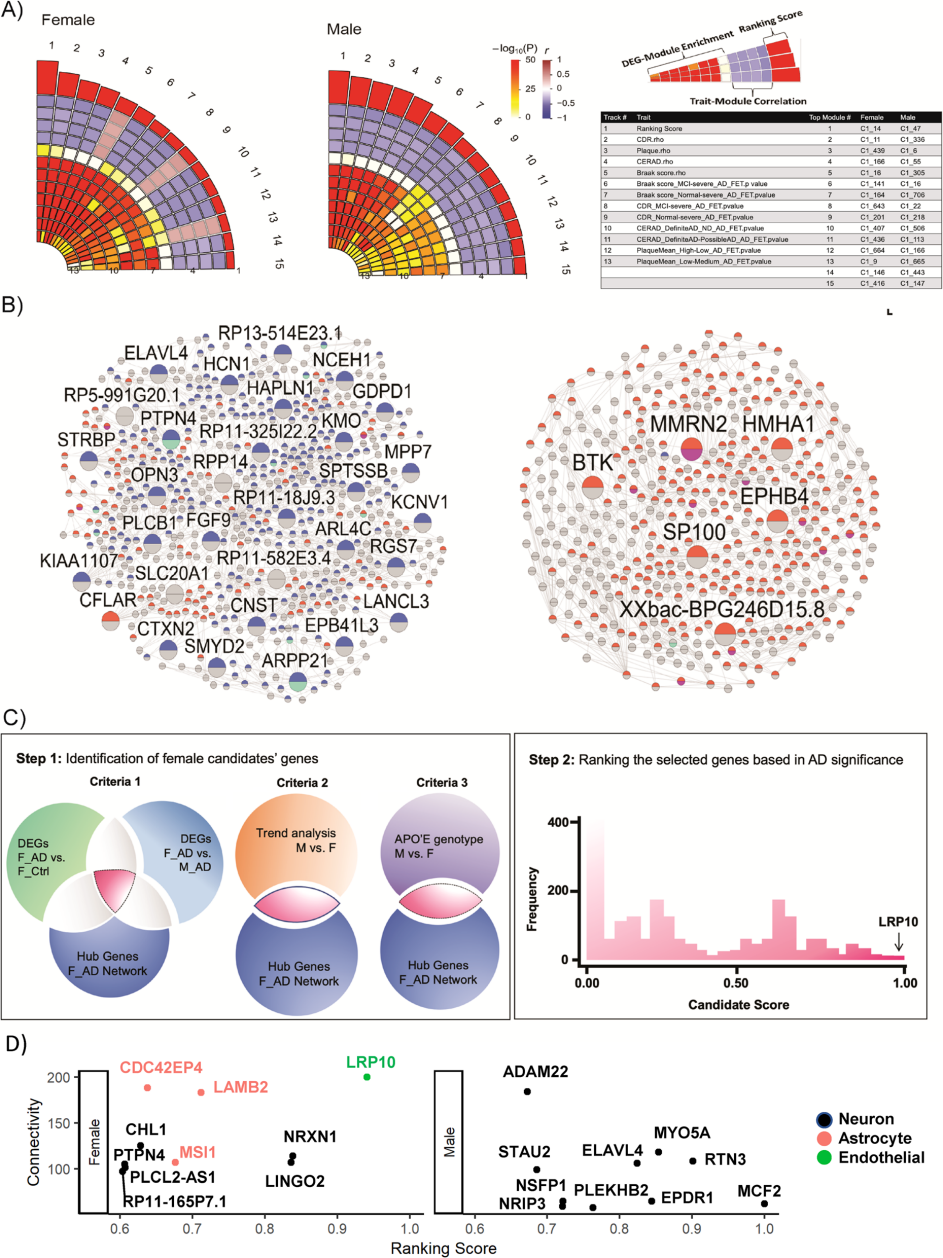
Sex-specific Co-expression Network Modules of AD Para-hippocampal Brain Region. **A** The top 15 MEGENA modules (with specific module number provided in the table) most associated with AD, which were most enriched for DEGs identified between AD and the control as well as significantly correlated with AD clinical traits with multiple tracks illustrating the different properties of the modules, such as strength of correlation between modules and the neuropathological/cognitive traits and significance of module enrichment for TCGs. The table showed the traits for the tracks #1–13 (the outmost track is the overall score). The tracks #2–5 corresponded to the correlations between a module and four traits including CDR, Plaque Density, CERAD, and Braak stage. Tracks #6–13 corresponded to the enrichment of DEG signatures (based on the comparisons including Medium vs High Braak stage, Low vs High Braak stage, MCI versus AD by CDR, Normal control versus AD by CDR, Definite AD versus Normal Control by CERAD, Definite AD versus Possible AD by CERAD, High versus Low Plaque density, Medium versus Low Plaque density) in modules. **B** Modules that were most enriched for neuron (left) and microglia (right) marker genes in the female AD network. The pie chart of each node indicates whether it was a DEG identified between AD and the control (upper half) or between women and men (lower half). Warm colors in the pie chart represented the upregulation of the gene in AD/women; cool colors in the pie chart represented the downregulation of the gene in AD/women. Nodes with large sizes and labels were sex-biased AD-associated candidate genes identified in this study. **C** Procedure for identification of KND genes for female and male AD. A summary score for each KND gene was calculated based on multiple *p* values derived from module-traits correlation and module-DEG enrichment analyses. LRP10 was identified as the top female KND candidate gene in the PHG from the MSBB cohort using the most stringent selection criteria (Criterion 1) with the highest rank score of 0.94 among all female KND candidate genes (the range of 0–1). **D** The top KND genes for female AD (left) and male AD (right). These candidates had high network connectivities (y-axis) and high summary scores (x-axis) (Supplemental Tables 10–11)

The Multiscale Embedded Gene co-expression Network Analysis (MEGENA) [25] was performed on the brain-region- and sex-specific RNA-seq data to identify co-expression gene modules reflecting coherent biological activities in each brain region (PHG and PFC) in each sex group during AD development. Supplemental Fig. 5A shows the female and male specific gene co-expression networks in the PHG with nodes colored by module membership. The module assignment for each gene in each brain region can be found in Supplemental Table 4 and two example gene modules were shown in Supplemental Fig. 5B. The functional pathways most enriched in the modules of both female and male AD networks from the PHG region in the MSBB cohort include immune system process, nervous system development, oxidative phosphorylation and neurodegenerative disease (i.e., AD, Parkinson's disease and Huntington’s disease) pathways (Supplemental Fig. 6; Supplemental Table 5). Modules were then ranked based on multiple features including module-trait correlations and modular enrichment for the TCG signatures (Supplemental Table 6). Figure 2A shows the features of 15 top-ranked modules of both female and male networks in the MSBB cohort. Modules highly correlated with AD clinical and pathological traits were of major interest. Cell type components of each module were determined by the enrichment of brain cell-specific gene signatures [10]. Most of the top-ranked modules were mostly enriched for the neuronal and microglia-specific marker signatures (Fig. 2B; Supplemental Table 7). In both the female and male networks, the top AD associated neuronal modules (e.g., M14, M439 and M166 in the female network) were negatively correlated with clinical and pathological traits like clinical dementia rating (CDR), plaque burden and Braak stage etc., consistent with their enrichment of the down-regulated genes in AD while the astrocytic, endothelial and microglial modules (e.g., M16, M201, M483) were positively correlated with these traits, consistent with their enrichment of the up-regulated genes in AD (Supplemental Tables 6–7). Hub genes in the co-expressed gene modules were also identified by network connectivity (Supplemental Table 8).

The modular differential connectivity (MDC) analysis of the co-expression networks in both the PHG and PFC regions [28] was carried out to quantitatively compare network reorganizations (i.e., connectivity) in female and male network modules, with MDC > 1 indicating a gain of connectivity (GOC) and MDC < 1 suggesting loss of connectivity (LOC). The female gene modules maintained high GOC in comparison with the respective male ones, whereas the male gene modules showed either no connectivity changes or LOC in comparison with the female ones, suggesting sex-biased differences in gene co-regulation in AD. In the PHG region, 72% of the female AD modules showed significant GOC but none had LOC, whereas only 15% of male AD modules had significant GOC and 4% showed significant LOC (Supplemental Table 9). Similar connectivity patterns were observed in the PFC region, in which 65% of the modules showed GOC and none had LOC, whereas none of the male AD modules showed GOC and 19% showed LOC (Supplemental Table 9). Interestingly, the second-ranked module (M11) in the female AD network of the PHG region was associated with mitochondrial function which had a gain of connectivity (GOC) (MDC = 1.31, false discovery rate (FDR) = 0.04) when compared to the male network. On the contrary, the 6^th^ ranked module (M16) in the male network of the PHG was also involved in mitochondrial function but had a loss of connectivity (LOC) (MDC = 0.97, FDR < 0.01), suggesting sex-biased differences in mitochondrial perturbation which may contribute to disease pathogenesis.

### Identification of sex-specific key drivers in AD co-expression networks

Intramodular hub genes, the highly connected hub nodes in a gene network, have been shown to regulate other genes in the network and termed as key network drivers (KNDs) [42]. This approach has been successfully employed to identify novel pathways and key driver genes of complex human diseases such as AD [22], Parkinson’s disease (PD) [43], melanoma [44] and gastric cancer [45] and notably the top drivers predicted for the abovementioned diseases were experimentally validated in vitro or in vivo. By definition, a driver gene for AD should regulate a number of other genes related to AD, therefore, a candidate sex-specific driver of AD should be a driver of a module associated with AD in a sex specific gene co-expression network. We further defined that a sex specific candidate driver should fit into any of these three criteria: Criterion 1—differential expression in mRNA between AD and control and between females and males in AD; Criterion 2 -opposite expression trends between females and males in at least two clinical traits; Criterion 3—opposite expression trends between females and males across *APOE* genotypes. Identification of sex specific key drivers of AD utilized three complementary strategies including differential expression analysis (DEA), trait-based differential trend analysis (TDTA) and *APOE* genotype based differential trend analysis (ADTA). Key network driver genes that meet any of the above three criteria were selected and further rank-ordered by the strength of association with AD (Step 1 in Fig. 2C).

In the PHG of the MSBB cohort, 5,135 KNDs for 726 modules were identified in the female AD network and 5245 KNDs in 878 modules of the male AD network (Supplemental Table 10). In the PFC of the ROSMAP cohort, 3,196 KNDs for 355 modules were identified in the female AD network and 2,623 KNDs in 279 modules of male AD network (Supplemental Table 10). Three complementary strategies including DEA, TDTA and ADTA identified 295 female-specific and 225 male-specific KNDs in the PHG region, and 478 female-specific and 867 male-specific KNDs in the PFC region (Supplemental Tables 10 and 11). Most of the sex-specific KNDs were selected by ADTA, which identified KNDs with opposite expression trends in *APOE* genotypes between female *versus* male. These results suggest that *APOE* genotypes play a critical role in driving sex differences in AD gene expression. Based upon brain cell-type specific gene signatures in each module, we assigned the brain cell type of each module for its member gene (Supplemental Tables 4 and 7). About 47% (350) of the 751 female-specific KNDs were neuronal genes (FET *p* = 2.48E-43, 1.95-fold enrichment (FE)) while 41% of the 1068 male-specific KNDs were neuronal genes (FET *p* = 1.99E-19, 1.44 FE) (Supplemental Tables 10 and 11). Fifty-two sex-specific KNDs of AD were also known AD genetic risk factors based on the AlzGene database and the genes identified from a large-scale meta-analysis carried out by the International Genomics of Alzheimer’s Project (IGAP) [46, 47]. In addition, the PHG and the PFC shared 23 female-specific AD KNDs and 25 male-specific AD KNDs. Nine AD KNDs were commonly shared between both sexes and both brain regions.

**Fig. 3:**
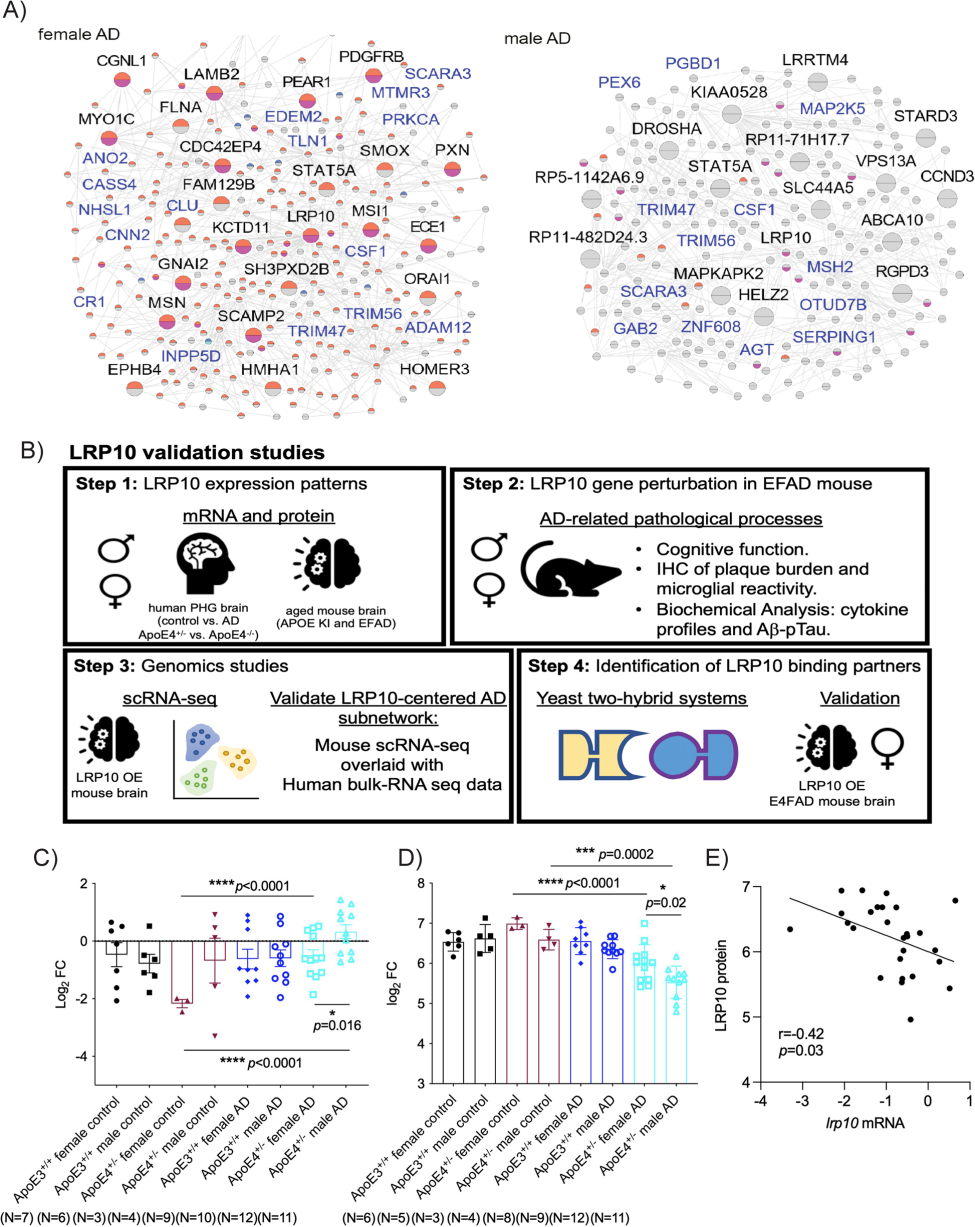
LDLR-related protein 10 (LRP10) Identified and Validated as a Sex-specific Key Regulator of AD. **A** LRP10-centered 2-layer networks constructed with the female AD samples (Left) and the male AD samples (Right). The pie chart of each node indicates whether it is a DEG identified between AD and the control (upper half) or between women and men (lower half). Warm colors in the pie chart represent the upregulation of the gene in AD/women; cool colors in the pie chart represent the downregulation of the gene in AD/women. Genes with blue labels are AD risk genes from Alzgen. **B** Procedure of functional validation of LRP10. Levels of **C** *lrp10* mRNA by qPCR analysis (data presented as log_2_FC) and **D** LRP10 protein by western blot (data presented as log_2_FC) were compared between AD *versus* control, male *versus* female, APOE4 carriers (APOE4^+/-^) *versus* non carriers (APOE3^+/+^) in the PHG human brain samples. ANOVA with post-hoc tests to determine group differences for multiple comparisons and independent-samples *t*-tests for paired comparisons. ****p < 0.0001; ***p < 0.001; *p < 0.05. **E** The correlation of qPCR analysis (*lrp10* mRNA) *versus* western blot analysis (LRP10 protein) of the PHG human samples were shown

Sex-specific KNDs were then rank-ordered by the strength of association with AD (Step 2 in Fig. 2C), based on the hypothesis that expression patterns of the genes closely connected with the KNDs in the co-expression network should be consistent with those of the KNDs determining or influencing a phenotype. The association of KNDs with AD was evaluated by the enrichment of the DEGs, which were detected between female AD *versus* female control, male AD *versus* male control, or female AD *versus* male AD, in the 3-layer co-expression networks centered around a candidate gene (female AD networks for female candidate genes and male AD network for male candidate genes). The rank order was calculated based on multiple *p* values calculated from module-traits correlation and module-DEG enrichment analyses (Supplemental Tables 10 and 11). Among candidate genes, *LRP10* was ranked as a top KND of the female AD network with a score of 0.941 (the range of score is between 0 (the least important) and 1 (the most important; Fig. 3A; Fig. 2C and D; Supplemental Tables 10–11) and thus was prioritized for validation studies. It should be noted that *LRP10* was selected from the female KND genes of the MSBB cohort that fit the most stringent selection criteria (Criterion 1: candidate genes with differential expression in mRNA between AD *versus* control and between females *versus* males in AD; Supplemental Table 10), whereas the majority of male candidate KND genes were identified from the ROSMAP that fit the least stringent selection criteria (Criterion 3: candidate genes with opposite expression trends between females *versus* males across *APOE* genotypes; Supplemental Table 11).

### LDLR-related protein 10 (LRP10) was validated as a sex-specific key regulator of AD

In the *LRP10*-centered female AD co-expression network (Fig. 3A), several sex-specific AD KNDs were closely connected to *LRP10* with similar expression patterns, i.e., up-regulated in female AD groups. In addition, many neighboring genes of *LRP10* in the co-expression network were known AD risk genes, such as *CHST3*, *CLU*, *CR1*, *CHD4*, *ADAM12*, and *CSF1*, suggesting the involvement of *LRP10* in AD pathogenesis. Figure 3B then provided a summary of studies to validate the role of LRP10 as a potential sex-specific key regulator of AD. From qPCR analysis, *lrp10* mRNA was significantly increased in female *APOE4*^+/-^ AD brains (PHG) compared to female *APOE4*^+/-^ controls with no significant differences in male *APOE4*^+/-^ AD *versus* male *APOE4*^+/-^ controls (Fig. 3C and Supplemental Table 12; *APOE4*^+/-^ female AD *versus APOE4*^+/-^ female control: log_2_FC -0.52 *versus* -2.17, *p* < 0.0001; *APOE4*^+/-^ male AD *versus APOE4*^+/-^ female control: log_2_FC 0.32 *versus* -2.17, *p* < 0.0001; *APOE4*^+/-^ male AD *versus APOE4*^+/-^ male control: log_2_FC 0.32 *versus* -0.68, *p* = 0.28). *Lrp10* mRNA was lower in *APOE4*^+/-^ female AD brains when compared to *APOE4*^+/-^ male AD counterparts (Fig. 3C and Supplemental Table 12; *p* = 0.016). On the other hand, LRP10 protein levels were significantly decreased in both female and male *APOE4*^+/-^ AD brains of the PHG region when compared to normal aged controls (Fig. 3D and Supplemental Table 12), which are consistent with previous studies [48]. However, levels of LRP10 protein were higher in *APOE4*^±^ female AD brains when compared to *APOE4*^+/-^ male AD counterparts (Fig. 3D and Supplemental Table 12; log_2_FC 6.00 *versus* 5.53, *p* = 0.02). There was a reciprocal correlation between *lrp10* mRNA and LRP10 protein expression (Fig. 3E; *r* = -0.42, *p* = 0.03), suggesting possible post-translational modifications such as an accelerated LRP10 protein degradation with a compensatory up-regulation of transcriptional machinery.

It should be noted that sex differences in *lrp10* mRNA and LRP10 protein expression were only seen in *APOE4*^+/-^ AD subjects (*APOE4*^+/-^ female AD with lower *lrp10* mRNA levels and higher LRP10 protein levels when compared to *APOE4*^+/-^ male AD; Fig. 3C and D). When combining data of *APOE4*^*−/−*^ and *APOE4*^+/-^ subjects together, sex differences in *lrp10* mRNA and LRP10 protein expression in AD samples become less evident (Supplemental Figs. 7A and 7B; *APOE4*^+/-^ female AD versus *APOE4*^+/-^ male AD: *lrp10* mRNA *p* = 0.11; LRP10 protein *p* = 0.07). Together, these results suggest sex-and APOE genotype-specific changes in LRP10 expression in AD.

### Characterization of AD-related phenotypes in EFAD mice with LRP10 over-expression

Similar reduction of LRP10 protein expression was seen in the hippocampal brain region of 6-month-old male and female *APOE4*^+*/*+^ mouse models with 5xFAD background (E4FAD in abbreviations)  when compared to the levels in littermates of male and female *APOE4*^+/+^ mice without 5xFAD background (Supplemental Fig. 7C). We next determined if over-expressing LRP10 could rescue cognitive dysfunction and AD-related pathologies in vivo using male and female EFAD mice.

**Fig. 4:**
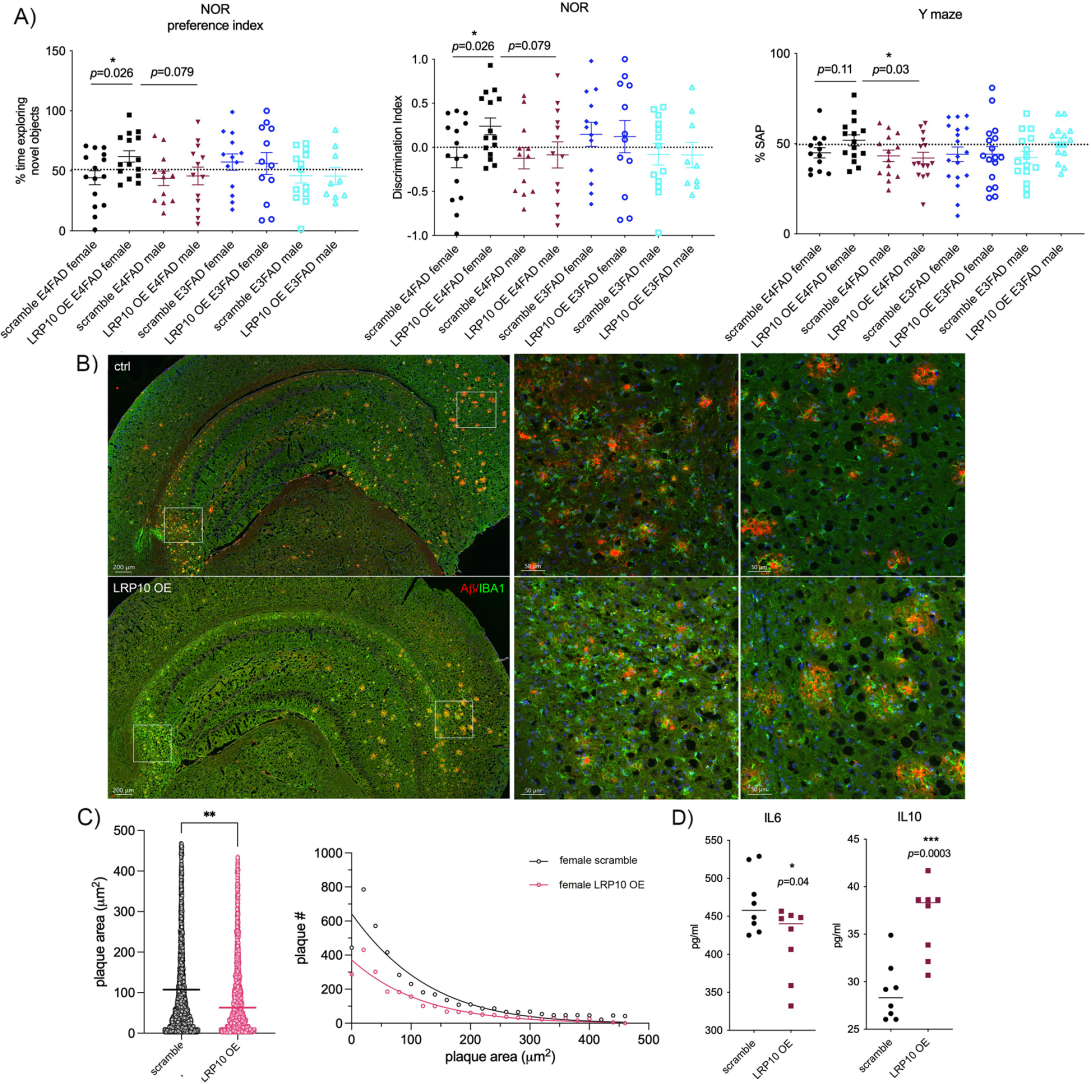
Characterization of AD-related Phenotypes in EFAD mice with LRP10 Over-expression (OE). **A** Novel Object Recognition (NOR) Studies: Preference index = (time exploring novel object)/(time exploring novel object + time exploring familiar object) and discrimination index = (time exploring novel object- time exploring familiar object)/(time exploring novel object + time exploring familiar object) in 8 groups of mice: scramble E4FAD female, LRP10 OE E4FAD female, scramble E4FAD male, LRP10 OE E4FAD male, scramble E3FAD female, LRP10 OE E3FAD female, scramble E3FAD male, and LRP10 OE E3FAD male. *N* = 9–15/group; *p < 0.05 with ANOVA tests. Y maze studies in 8 groups of mice: % spontaneous alternation percentage (SAP) = ({spontaneous alternation/(total number of arm entries -2)} × 100). *N* = 14–18/group; *p < 0.05 with ANOVA tests. **B** A representative image of brain section was shown with top panels scramble E4FAD female mouse brain and bottom panels LRP10 OE E4FAD female mouse brain (red: amyloid plaque staining; green: IBA1^+^ microglia). **C** Quantification of amyloid plaque burden in E4FAD female mouse hippocampus by density was measured by the size of all plaques (plaque area in mm^2^) in the brains of scramble (black scatter plot, each dot representing individual plaque) *versus* LRP10 OE (pink scatter plot) female E4FAD mice. Distribution of plaques measured by numbers of plaques in different sizes was compared between scramble *versus* LRP10 OE female E4FAD mouse brains as well. **D** Levels of IL6 and IL10 were compared between scramble *versus* LRP10 OE female E4FAD mice and data were presented as % of controls with the average of scramble E3FAD male mouse brain levels as 100%. *N* = 3–5/group; *p < 0.05 **p < 0.01 ***p < 0.001 by unpaired T-tests with Welch’s corrections

It was previously demonstrated that both male and female E4FAD or *APOE3*^+*/*+^ mouse models with 5xFAD background (E3FAD) manifested with AD-related pathological, neuro-inflammatory, and behavioral phenotypes at 4–8 months of age [29, 30], such as memory impairments measured by novel object recognition (NOR) tests with an inability to discriminate between novel and familiar objects [49]. Here we found that over-expression (OE) of LRP10 in female E4FAD mice rescued cognitive deficits when compared to scramble control counterparts (Fig. 4A, preference index: 43.5% *versus* 62.0%, *p* = 0.026). However, no statistically significant differences were seen between scramble *versus* LRP10 OE male and female E3FAD mice as well as male E4FAD mice. Moreover, the discrimination index studies using the differences in exploration times for novel *versus* familiar object [49] showed consistent results suggesting that impaired discrimination behaviors in female *E4FAD* mice were completely rescued by LRP10 OE (Fig. 4A, discrimination index: -0.13 *versus* 0.24, *p* = 0.026). The total amount of exploration time was comparable among all groups (data not shown). In addition, the Y-maze spontaneous alternation test was carried out to evaluate cognitive functions and learning in mice [50, 51]. Again, female E4FAD mice with LRP10 OE showed improvements in percentage of alteration when compared to scrambled controls or male E4FAD mice with LRP10 OE (51.1% *versus* 43.5% and 42.0%, respectively; female E4FAD LRP OE *versus* scramble control *p* = 0.11 and female *versus* male E4FAD LRP OE *p* = 0.03). No statistically significant differences were seen between scramble and LRP10 over-expressing male E4FAD, male E3FAD mice or female E3FAD mice.

Furthermore, immunohistochemical analysis of amyloid plaque load and microglia migrated around plaques showed reduced plaque burden in female E4FAD mice with LRP10 OE when compared to control (Fig. 4B). The plaque quantification analysis showed a 58.6% reduction in mean plaque area measured by μm^2^ with LRP10 OE when compared to control (Fig. 4C; *p* = 0.008). The cumulative plaque distribution analysis indicated a more dramatic reduction in plaques smaller than 200μm^2^ with LRP OE (Fig. 4C). In addition, there was a significant increase in IBA1^+^ microglia recruited around amyloid plaques in female E4FAD LRP10 OE mice when compared to control counterparts (Fig. 4B; 41.5% increase, *p* = 0.048) with consistent increases in levels of anti-inflammatory cytokine IL10 (Fig. 4D; 26.3% elevation *p* = 0.003, respectively), and a reciprocal reduction in levels of proinflammatory cytokine IL-6 (Fig. 4D; 10.9% decrease, *p* = 0.04).

The biochemical analysis of female E4FAD mouse hippocampal brain regions further indicated that LRP10 OE (Supplemental Fig. 8A: 68.4% increase in LRP10 expression) significantly reduces levels of total Tau (Supplemental Fig. 8B: 49.6% reduction, *p* = 0.003), oligomer Aβ_42_ (Supplemental Fig. 8C: 2.60 *versus* 2.22 pg/ml, *p* = 0.04), soluble Aβ_42_ (65.9 *versus* 75.7 pg/ml, *p* = 0.03) and Aβ_40_ (154.4 *versus* 175.9 pg/ml, *p* = 0.003), with a trend of reduction in pTau levels (Supplemental Fig. 8B: 46.4% reduction, *p* = 0.20) when compared to scramble controls. No significant differences were seen in APOE levels between scramble *versus* LRP10 over-expressing female E4FAD mice (Supplemental Fig. 8C). No significant changes in total numbers of IBA1^+^ microglia in female E4FAD mice with LRP10 OE (Supplemental Fig. 8D), as well as in TNFα or IL17 levels (Supplemental Fig. 8E).

On the other hand, the immunohistochemical and biochemical analysis of male E4FAD mouse hippocampal brain regions suggested that LRP10 OE did not induce any statistically significant changes in IL6, pTau, soluble Aβ_42_ or Aβ_40_ levels (Supplemental Figs. 9A, 9B and 9C). There was a reduction in the total tau levels (Supplemental Fig. 9B: 49.3% reduction of total tau, *p* = 0.02) with an increase in IL10 levels (Supplemental Fig. 9A: 19.3% increase, *p* = 0.03). However, there was an increase in oligomer Aβ_42 _levels (Supplemental Fig. 9C: oligomer Aβ_42_ 1.59 *versus* 2.04 pg/ml, *p* = 0.02) as well as amyloid plaque burden in male E4FAD mouse brains (Supplemental Fig. 9D: an increase in mean plaque area in male LRP10 OE when compared to control with *p* < 0.0001), which may explain no functional rescue in these animals. Overall, these results suggest that factors other than LRP10 may contribute to the pathogenesis of male ApoE4^+/-^ AD subjects.

Together, these results support the notion that LRP10 is a causal regulator for AD, whose expression is significantly associated with cognition performance and development of AD pathology in sex- and APOE genotype-specific manners.

### Cell-type specific changes in LRP10 OE mouse brain

**Fig. 5:**
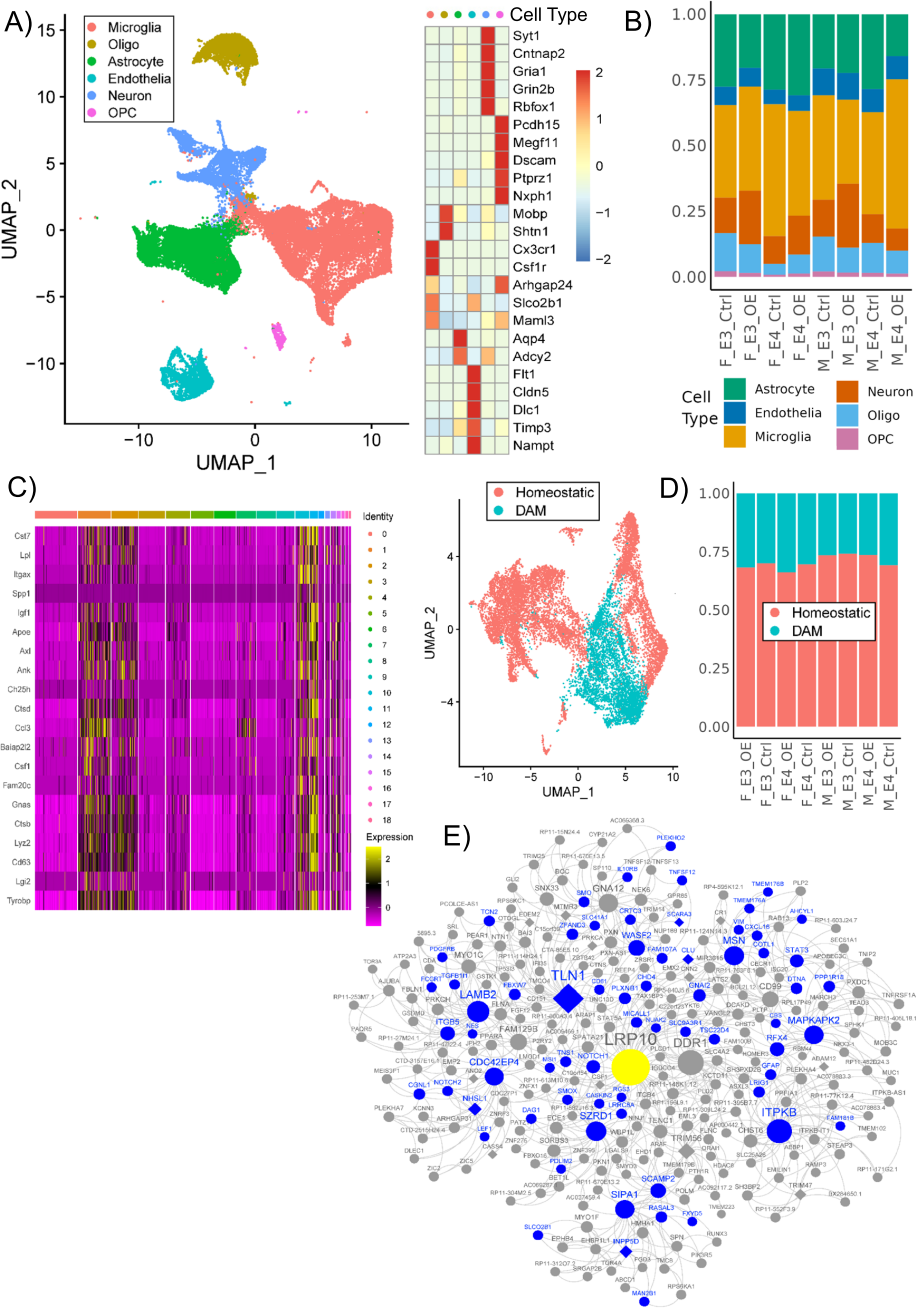
Cell-type Specific Changes in the LRP10 OE Mouse Brains. **A** UMAP visualization showing clustering of single cells (left) and expression patterns of the cell type marker genes in each cell type (right). **B** Cell type proportion analysis for six brain cell types in each experimental group. **C** Microglia subtypes were identified using the DAM marker genes (left). UMAP visualization showing clustering of homeostatic *versus* DAM subclusters (right). **D** Microglial subtype proportion analysis in each experimental group. **E** LRP10-centered gene co-expression network in the female AD human brains was enriched with DEGs identified between LRP10 OE *versus* control female E4FAD mouse brains. Blue nodes were the DEGs between female E4FAD mice with LRP10 OE *versus* control conditions. Diamond Nodes were the AD risk genes identified from previous GWAS studies

To better understand downstream signaling pathways mediated by LRP10 in female *APOE4*^±^ AD brains, we next performed a comprehensive mapping of brain cell populations in LRP10 OE *versus* scramble control EFAD mouse brains based on single cell RNA-sequencing (scRNA-seq) of the hippocampal samples. After QC of the scRNA-seq data (see methods for details; Supplemental Fig. 10), the clustering analysis identified 6 major brain cell types including neuron, astrocyte, microglia, oligodendrocyte, oligodendrocyte progenitor cell (OPC), and endothelial cell (Fig. 5; Supplemental Fig. 11). Results suggested an increased proportion of neurons seen with LRP10 OE in female E4FAD, as well as both male and female E3FAD mouse brains when compared to control counterparts, but not in male E4FAD mice (Fig. 5A-B and Supplemental Table 13; 40.3% increase in female E4FAD but 12.6% decrease in male E4FAD). On the other hand, the microglial proportion was reduced in female E4FAD mouse brains but increased in male E4FAD mouse brains with LRP10 OE (Fig. 5B and Supplemental Table 13; 20.5% decrease in female E4FAD but 46.2% increase in male E4FAD). Similarly, opposite trends of changes in astrocyte and oligodendrocyte proportions were seen in female *versus* male E4FAD mouse brains with LRP10 OE (Fig. 5B and Supplemental Table 13).

Sub-clustering of the microglial population identified homeostatic (HAM) *versus* damage-associated microglia (DAM) sub-clusters based on marker gene signatures (Fig. 5C, Supplemental Figs. 11C-D). There were opposite cluster proportion changes with LRP10 OE between female and male E4FAD mice (Fig. 5D and Supplemental Table 13). LRP10 OE expanded the portion of DAM in female E4FAD mice (11.4% increase compared to ctrl), consistently with observations of increased IBA1^+^ microglia recruited around amyloid plaque in this condition (Fig. 4B). In contrast, LRP10 OE reduced the DAM portion in male E4FAD (14.1% decrease). The sub-clustering analysis was also applied to the neuronal clusters and identified 4 neuronal subtypes including Glutaminergic neurons, Dopaminergic neurons, and two subtypes of GABAergic neurons (GABAN-T1 and GABAN-T2) (Supplemental Fig. 12; Supplemental Table 14A-B). LRP10 OE reduced GABAN-T1 in female E3FAD by 2.8%, male E3FAD by 3.8%, and male E4FAD by 5.5% but increased GABAN-T1 in female E4FAD by 5.7%. On the other hand, LRP10 OE reduced GABAN-T2 in female E3FAD by 18% but increased GABAN-T2 in male E4FAD by 10% while the changes in female E4FAD and male E3FAD were minimal (< 1%). The impact of LRP10 OE on dopaminergic neurons was more dramatic. LRP10 OE increased dopaminergic neurons in female E3FAD by 19.6% and in male E3FAD by 7.3% but reduced dopaminergic neurons in male E4FAD by 12%. These results demonstrated the impact of *LRP10* on brain cell populations was sex- and APOE genotype-dependent.

The scRNA-seq data from the EFAD mouse *LRP10* OE experiments allowed us to identify sex-specific targets of *LRP10* which were then intersected with the respective sex-specific, *LRP10*-centered co-expression subnetworks constructed from the bulk RNA-seq data in the PHG in the MSBB cohort. Differentially expressed genes between *LRP10* OE and control were identified for each of the five major brain cell types in each sex- and APOE-genotype group. Supplemental Fig. 13 showed the functional enrichment pathways of these DEG signatures. The down-regulated DEGs in astrocytes from female E4FAD groups had most enriched pathways including responses to stress, regulation of cell killing and cell communication, etc. The female-specific *LRP10* targets, which were derived as a union of the DEG signatures in the five brain cell subtypes in the female E3FAD and E4FAD mouse cohorts, were significantly enriched in the *LRP10*-centered, L-layer subnetworks of the female AD human brains (L = 4: adjusted *p* = 1.46E-06, fold enrichment (FE) = 1.38; L = 3: adjusted *p* = 1.21E-07, FE = 1.5; L = 2: adjusted *p* = 2.9E-04, FE = 1.8; Fig. 5E). However, the male mouse specific *LRP10* targets were not significantly enriched in any *LRP10*-centered subnetworks in male AD human brains. The data further validated our prediction of *LRP10* as a key network regulator of AD in females but not in males.

We also investigated the impact of *LRP10* OE on transcriptomic profiles in the microglial and neuronal subtypes. LRP10 OE induced more gene expression changes in DAM than in homeostatic microglial subclusters (Supplemental Table 15) and the multi-intersection analysis showed that within DAM or homeostatic microglial subcluster, each sex- and ApoE-genotype group had unique DEGs with significant overlaps across different groups and many in opposite directions (Supplemental Fig. 14A-B). As shown in Supplemental Table 14C, most DEGs induced by LRP10 over-expression were observed in GABAN-T1 from female and male E4FAD as well as in Glutaminergic neurons from male E3FAD and male E4FAD. Supplemental Fig. 12C further showed that the *LRP10* over-expression induced transcriptomic changes in GABAN-T1 from female E4FAD significantly overlapped with those from male E4FAD but many of them were in opposite directions. The transcriptomic analysis further demonstrated the differential effects of *LRP10* on males and females in the context of AD.

Together, these results suggest that *LRP10* impacts brain cell populations in sex- and APOE genotype-specific manners, with neurons and microglia as the most affected cell types.

### Identification of LRP10 binding partners

**Fig. 6:**
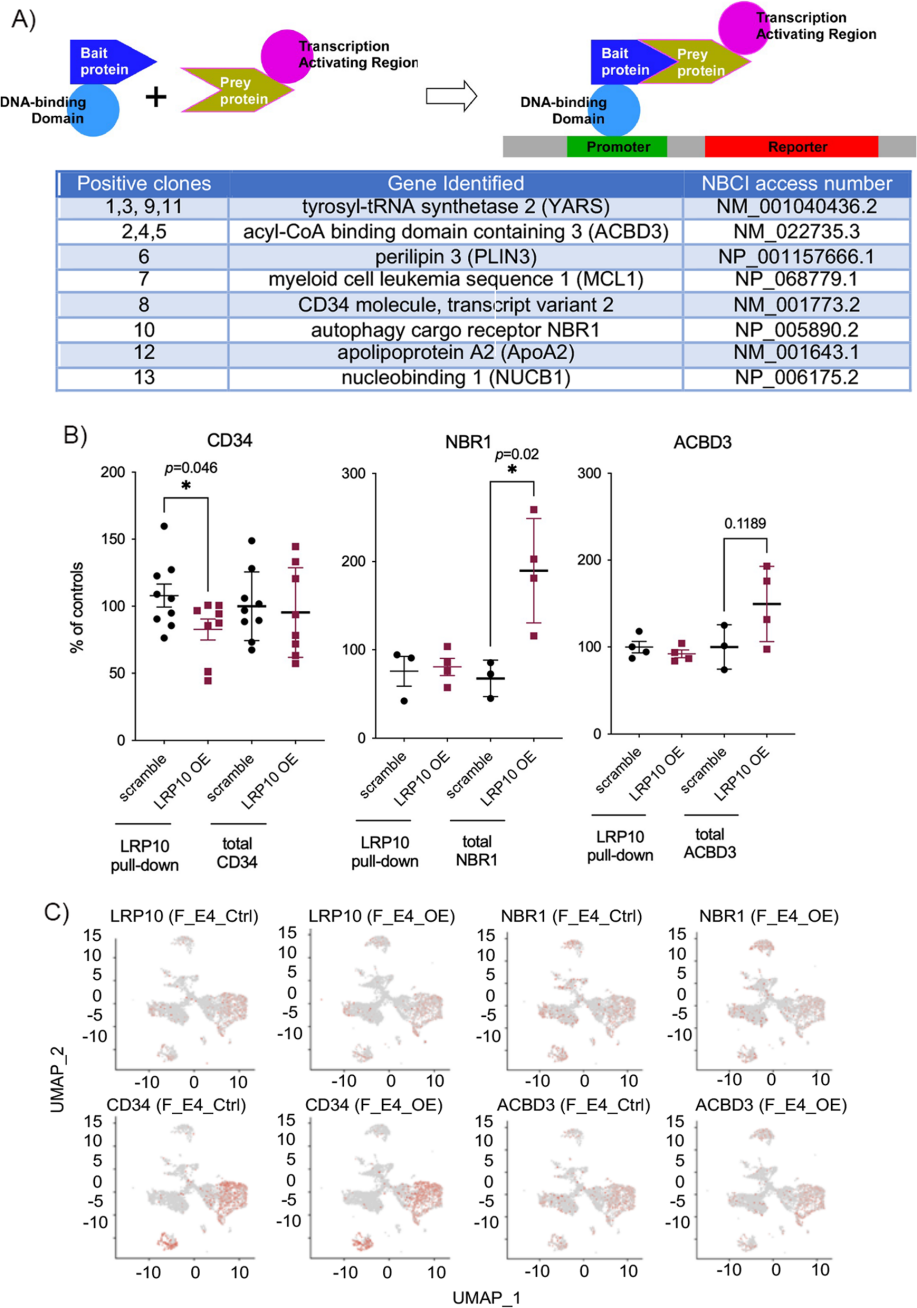
LRP10 Binding Partners. **A** Left: The design of the yeast two-hybrid system screening assays. Right: The summary table of eight positive hits that were identified and validated by β-galactosidase assays. **B** The interaction between LRP10 and its binding partner CD34, NBR1 or ACBD3 was detected by co-immunoprecipitation (co-IP) pull-down in female E4FAD mouse brains of LRP10 OE *versus* scramble controls. The amounts of total input (CD34, NBR1 and ACBD3) in mouse brain lysates were determined as well. **C** Cell type specific enrichment expression patterns of LRP10 and its binding partners (CD34, NBR1 and ACBD3) in female E4FAD mouse brains of LRP10 OE *versus* scramble controls

To identify novel LRP10-interacting proteins, LRP10 cytoplasmic tail was used as a bait to screen an adult human brain cDNA library using the yeast two-hybrid system. A total of 13 positive clones were identified and validated by β-galactosidase assays (Fig. 6A and Supplemental Fig. 15A). The subsequent sequence of positive clones and further validation by β-galactosidase assays led to eight unique positive hits including acyl-coA binding domain containing 3 (ACBD3), nucleobindin 1 (NUCB1, also known as calnuc), autophagy cargo receptor (neighbor of BRCA1 gene 1 variant 1, NBR1-v1), tyrosyl-tRNA synthetase 2 (YARS2), perilipin 3 (PLIN3), apolipoprotein A2 (ApoA2), myeloid cell leukemia 1 (MCL1) and CD34 (Fig. 6A).

The interaction of LRP10 with its putative binding partners such as CD34, NBR1 and ACBD3 were further studied in female E4FAD mouse brains of LRP10 OE *versus* controls by co-immunoprecipitation (co-IP) methods. The amount of CD34 interacted with LRP10 was significantly reduced in the LRP10 OE mouse brains when compared to controls without any changes in total protein levels (Fig. 6B). On the other hand, there was a significant increase in NBR1 total protein levels and a trend of increases in ACBD3 total protein levels without any significant changes in the amounts of NBR1 or ACBD3 that interacted with LRP10 in the LRP10 OE female E4FAD mouse brains when compared to controls (Fig. 6B).

The scRNA-seq data allowed us to further examine brain cell type enrichment of LRP10 and its binding partners. While LRP10 was enriched in brain endothelial and microglial clusters, its binding partners such as CD34, NBR1 and ACBD3 were highly enriched in these brain cell type clusters as well (Fig. 6C and Supplemental Figs. 15B-C), suggesting potential roles for the interaction of LRP10 and its binding partners in regulating brain cell-type specific functions. Future studies will dissect the functional relevance of the interaction between LRP10 and its binding partners in brain microglia and endothelial cells in AD pathogenesis.

It should be noted that no significant changes were detected in protein expression levels of other LRP receptors such as LRP1, LDLR, or LRP3 with a trend of reduction in LRP6 in the LRP10 OE E4FAD mouse brains when compared to controls (Supplemental Fig. 15D, LRP6: 17% decrease, *p* = 0.14).

In summary, our data provide mechanistic insights into putative downstream signaling pathway(s) mediated by LRP10 and its binding partners in specific brain cell types that may contribute to AD pathogenesis in female *APOE4*^+/-^ carriers.

## Discussion

Our study presented here is the first large-scale characterization of sex-specific gene expression regulation and network organization in AD. The pathogenesis of AD like many other complex diseases stems from perturbation of gene–gene interactions, and the functional roles of key driver genes of such diseases can only be better understood when taking into account molecular networks that define disease states. Gene network analysis provides such a tool that allows us to identify not only high level gene interaction and co-regulatory structures but also key network drivers of disease [52]. In this study, we used an integrative network analysis to identify sex-specific networks and gene targets of AD. Consistently with prior observations [53–55], the transcriptome-wide analysis showed that the PHG brain region had much more significant gene expression changes in the DE analyses compared to the PFC region (Fig. 1). Moreover, the top-ranked co-expressed gene modules in the PHG were associated with nervous system and immune responses (Fig. 2), which are consistent with prior studies [56, 57]. Interestingly, a commonly shared function of the modules across the PHG and PFC regions was oxidative phosphorylation with opposite connectivity changes between females and males by differential connectivity analysis. Previous studies have reported that mitochondria functions differently between women and men. In women, intact mitochondrial function protects individuals against Aβ toxicity probably due to estrogen-mediated suppression in ROS and apoptogenic signals generated in women [58]. For this very reason, women may suffer in a greater degree from mitochondrial dysfunction when estrogen levels are significantly reduced after menopause.

On the other hand, we also observed strong brain-region specific, sex-biased gene expression patterns in AD pathogenesis. Such brain-region specific, sex-biased patterns may arise from differences in vulnerabilities and/or resilience in different brain regions at different stages of AD development between females and males. For example, a recent study revealed the distinct architectures of tau-based brain-region connectivity networks in males and females, and further showed that the network architecture in women favored a more rapid spread of neurofibrillary tangles in the brain [59]. Moreover, the interplay among sex, genetic factors (e.g., APOE variants) and various environmental factors may contribute to brain-region specific, sex-biased gene expression patterns in AD. It has been shown that risk allele variants at the *APOE* locus have a stronger effect in females than in males [60]. Furthermore, the dynamics of sexually dimorphic hormones such as estrogen [61–63] may affect different brain regions disproportionally.

Among the prioritized candidate genes, *LRP10* is top-ranked for its high network connectivity, sex-specific differential expression in AD and *APOE4* dosage dependency. In our co-expression network constructed from female AD brains, 6 candidate genes (*MSI1*, *CDC42EP4*, *MSN*, *PXN, ESAM* and *CLU*) were direct neighbors of *LRP10* (Fig. 3). *CLU* also known as *apolipoprotein J* (*APOJ*), was identified as an AD risk gene by a large scale meta-analysis [46]. With its flexible structures, *CLU* is able to bind a variety of physiological ligands, including Aβ [64]. By binding to its major receptor - LRP2, the CLU-Aβ complex can then be eliminated from the brain [64]. The close relationship between *CLU* and *LRP10* revealed by the co-expression network suggests that like LRP2, LRP10 may also be involved in brain Aβ clearing through an LRP10-CLU/Aβ pathway. Female-specific AD KNDs such as *CDC42EP4*, *MSN*, *PXN,* and *ESAM* were closely related to *LRP10* in the female co-expression network in the PHG with similar expression patterns, suggesting that these cytoskeleton/adhesion-related genes may also participate in the Aβ trafficking and clearing through the same pathway. For example, MSN protein levels were significantly different between AD and control brains [65]. In addition, PXN was up-regulated in the hippocampus, superior frontal gyrus and post-central gyrus of AD brains, co-localizing with Aβ-containing plaques [66].

The LRP10 protein levels were previously shown to be significantly lower in the frontal cortex and hippocampus of AD brains than those in controls with a greater extent of reduction in female AD subjects than in male counterparts [48]. In our study, we demonstrated a significant increase of *lrp10* mRNA levels and a dramatic reduction of LRP protein expression in APOE4^+/-^ AD brains when compared to control counterparts (Fig. 3C and 3D) suggesting post-transcriptional and/or post-translational modifications such as a possibility of accelerated LRP10 protein degradation with compensatory up-regulation of transcriptional machinery. The discordance or decoupling in mRNA and protein expression of the same genes has been reported in studies with human aging [67] and neurodegenerative disorders such as tauopathy [68] and AD [69] with proposed mechanisms such as changes in expression profiles of RNA binding proteins and microRNAs [67], as well as post-translational modifications with changes in protein folding, degradation and/or half-lives [69–72]. Based on our findings, we are currently studying whether half-lives of *lrp10* mRNA and LRP10 protein are specifically altered in female APOE4^+/-^ AD brain cells when compared to other counterparts.

Intriguingly, sex differences in LRP10 expression can only be observed in APOE4^+/-^ AD subjects with *lrp10* mRNA levels lower in females than males AD subjects, whereas LRP10 protein levels were higher in females than males as suggested by our data (Fig. 3C and 3D). Consistently in gene perturbation in vivo studies, up-regulation of LRP10 expression showed beneficial effects only in female E4FAD mice but not in male E4FAD mice or E3FAD mice (Fig. 4A), supporting the notion that LRP10 as a causal regulator for AD, impacts cognition performance and development of AD pathology in sex- and APOE genotype-specific manners. Intriguingly, we also performed a separate set of experiments in female and male APOE3 and APOE4 KI mice without 5xFAD background as well as female and male wildtype mice without 5xFAD background with LRP10 knockdown (KD) treatment to determine if down-regulation of LRP10 could induce any worsening cognitive function in these mouse models. However, we did not observe any statistically significant difference between LRP10 KD *versus* control in these animals (Data not shown). It is possible that the beneficial effects of LRP OE on female E4FAD mice we observed (Fig. 4) may exhibit mainly through accelerated clearance of amyloid pathology, and that without amyloid pathology this impact is hard to be appreciated. Future studies are needed to further characterize the mechanisms of action induced by LRP10 in female APOE4^+/-^ AD brains. Together, these results further strengthen the needs of future drug development effort driven by precision-medicine and tailored by sex and APOE genotypes.

Furthermore, our scRNA-seq analysis of EFAD mouse brains after LRP10 over-expression indicated cell-type specific changes in neuron and microglia proportion (Fig. 5B and Supplemental Figs. 12–14) with consistent immunohistochemical data showing increased microglial recruitment around amyloid plaques (Fig. 4B). These results suggest potential functional roles of LRP10 in amyloid clearance through modulation of microglial function. LRP10 was previously implicated in APP trafficking and processing [48] with a subcellular localization in the trans-Golgi network (TGN), plasma membrane and endosomes. Over-expression of LRP10 in human neuroblastoma SH-Sy5y cells led to an accelerated APP transport from TGN to the plasma membrane with increased APP maturation and reduced Aβ production [48]. It is possible that in female APOE4^+/-^ AD brains, reduction in LRP10 protein expression may result in an increased Aβ production with impaired APP trafficking [48] as well as a reduced Aβ clearance by microglia (as supported by our data) leading to AD pathological changes. It would be important to dissect how sex and APOE genotype specifically affect LRP10 expression in certain brain cells during AD development.

Unlike other well-characterized APOE receptors like LRP1 and LDLR, LRP10 is identified as a distant family member of APOE receptors [73] with functional roles in AD, particularly less recognized in sex-specific AD development. Interestingly, a recent study identified several genetic variants of *LRP10* in familial Parkinson’s disease and dementia with Lewy bodies with possible loss-of-function effects on mRNA stability, protein stability and localization as potential pathogenic mechanisms [74]. It is possible that in APOE4^+/-^ female AD subjects, reduction of LRP10 protein is reminiscent of loss-of-function effects of *lrp10* in other neurodegenerative disorders. It would be interesting to determine if any loss-of-function genetic variants of *lrp10* can be identified in female APOE4^+/-^ AD subjects and how LRP10 modulates the development of AD in sex-specific manners in relation to other APOE receptors and/or downstream signaling pathways in future studies.

Intriguingly, our studies identified eight putative binding partners of LRP10 (Fig. 6). Among these positive hits, NUCB1 was the only one previously reported as a LRP10-binding protein and its interaction with LRP10 was shown to prevent the delivery of LRP10 to the lysosomes [75]. Our co-IP studies further demonstrated changes in the interaction between LRP10 and one of its binding partners CD34 with LRP10 OE, suggesting a possible involvement of this interaction in the LRP10-mediated function (Fig. 6B). The impact of sex and APOE genotypes on the interaction of LRP10 and its binding partners, CD34 in particular, as well as the expression levels of LRP10 binding partners such as NBR1 and ACBD3 will be further investigated in mouse brains with or without 5xFAD background in the presence or absence of perturbation of LRP10 expression (over-expression or knock-down). In addition, the scRNA-seq analysis identified brain cell type enrichment patterns of LRP10 and its binding partners in microglia and endothelial cells (Fig. 6C), consistent with the findings that LRP10 resides in the module enriched for endothelial and astrocyte marker genes based on the cell-type enrichment analysis of bulk RNA-seq analysis of human dataset (Supplemental Table 11). These results implicate putative roles in AD through regulation of these brain cell-specific functions. The initial transcriptomic analysis of sex and APOE-genotype specific changes in EFAD mouse brains with or without *LRP10* over-expression already indicates the distinct impacts of LRP10 on males and females as well as APOE genotypes in AD, validating the prediction of *LRP10* as one key molecular regulator of sex difference in AD based upon a highly integrated network biology analysis of two large-scale multi-omics cohorts in AD. We are currently analyzing available snRNA-seq data of human cohorts to parse out cell-type specific KNDs of female and male AD networks. The cross-examination of current findings from human bulk seq datasets with our ongoing in-depth analyses of human snRNA-seq datasets as well as the scRNA-seq data from the LRP10 OE experiments carried out in this study will provide a comprehensive and deep characterization of sex- and APOE-specific AD pathogenesis at the single cell level.

## Conclusions

In this study, we investigated transcriptome-wide gene expression and gene co-expression structures associated with sex-specific AD pathogenesis. We systematically analyzed brain samples collected from well-characterized individuals with the full spectrum of dementia and neuropathogenesis. Applying integrated systems biology approaches, we identified and validated a top candidate key driver gene *LRP10* with high confidence for further study. Gene perturbation studies of LRP10 expression in AD mouse brains revealed a functional role of LRP10 protein in AD pathogenesis in sex- and APOE genotype-specific manners. The findings of this study provide insights into key mechanisms mediating sex differences in AD and the role of interaction between sex and APOE genotypes in AD and will potentially facilitate the development of sex-specific treatment strategies. Further studies are needed to characterize the downstream signaling pathways of LRP10 through its interaction with its putative binding partners in LRP10-modulated APOE genotype-specific female AD pathogenesis.

## Supplementary Information

**Additional file 1: Supplemental Table 1.** Sample information for the human brain cohorts. The information of individual samples in the ROSMAPMSBBand cohorts was provided including sex, ApoE genotype, age of death, Braak score, plaque burdenor cognitive diagnostic score, CDRor MMSE, PMI and diagnosis based on CERAD and Braak scores. C) Summary statistics for various sex-ApoE-genotype subgroups.

**Additional file 2**: **Supplemental Table 2.** Differential expression analysis of the comparisons of various groups as the combinations of sex, disease status, and APOE genotype. Multiple tests were adjusted using the Benjamini–Hochberg’sFDR method. Genes with an FDR adjusted *p* value less than 0.05 and fold changegreater than 1.2 were considered significant. A) Summary of the numbers of DEGsfrom different comparisons in different brain regionsreported in 2B and 2C. B) The DEGs in the PHG of the MSBB cohort from the comparisons of sex specific and APOE genotype specific AD *versus* control subjects as well as disease status and APOE genotype specific males *versus* females. C) The DEGs in the PFC from the ROSMAP cohort from the comparisons of sex and APOE genotype specific AD versus control subjects as well as disease status and APOE genotype specific males *versus* females.

**Additional file 3**: **Supplemental Table 3.** Jonckheere Trend analysis to identify genes correlated with each clinical trait and APOE genotype in each sex in the PHG of MSBB and the PFC of ROSMAP. Multiple tests were adjusted using the Benjamini–Hochberg’s FDR method. Differences in trend between males and females were assessed as well. A-B) the PHG of MSBB cohort and C-D) the PFC of ROSMAP cohort. E) Intersection among the Jonckheere based DTG signatures in the PHG of the MSBB cohort. F) Intersection of the known AD risk genes and the Jonckheere based DTG signatures in the PHG of the MSBB cohort.

**Additional file 4**: **Supplemental Table 4.** Module assignment for the genes in the male and female gene coexpression networks in the PHG and PFC brain regions. A-B) the PHG of MSBB cohort and C-D) the PFC of ROSMAP cohort. 

**Additional file 5**: **Supplemental Table 5.** The top ranked modules and their enriched pathways in the male and female gene co-expression networks in the PHG and PFC brain regions. Fold enrichment and Fisher’s Exact Test based enrichment significance were reported. The top ranked 100 modules from A) female and B) male gene co-expression networks in the PHG brain regions, as well as the top ranked 50 modules from C) female and D) male gene co-expression networks in the PFC from the ROSMAP cohort.

**Additional file 6**: **Supplemental Table 6.** Correlations between clinical and pathological traits and gene modules in the male and female gene co-expression networks in the PHG of the MSBB cohort and the PFC of the ROSMAP cohort. Spearman’s rank correlation was used. Nominal correlation *p* values and corrected *p* values by the Benjamini–Hochberg’s method were reported for A) female and B) male gene co-expression network modules in the PHG brain region from the MSBB cohort, as well as C) female and D) male gene co-expression network modules in the PFC brain region from the ROSMAP cohort.

**Additional file 7**: **Supplemental Table 7.** Cell type specificity of gene modules in the male and female gene co-expression networks in the PHG of MSBB cohort and the PFC of the ROSMAP cohort. A) female and B) male AD subjects in the PHG of the MSBB cohort. C) female and D) male AD subjects in the ROSMAP cohort.

**Additional file 8**: **Supplemental Table 8.** Module hubs genes in the male and female gene co-expression networks in the PHG of MSBB cohort and the PFC of the ROSMAP cohort. A) female and B) male AD subjects in the PHG of the MSBB cohort. C) female and D) male AD subjects in the ROSMAP cohort.

**Additional file 9**: **Supplemental Table 9.** Modular differential connectivity analysis of male and female specific co-expression networks. MDC was calculated as the ratio of the mean module connectivity in one network to that for the same set of genes in another network. FDRs were calculated by two methods, sample-based permutation and gene-based permutation. The greater one of the two FDRs for each gene was taken as the final FDR for MDC of a module. The MDC analysis of A) female AD network modules *versus* male network in the PHG of the MSBB cohort, B) male AD network modules *versus* female network in the PHG of the MSBB cohort, C) female AD network modules *versus* male network in the PFC of the ROSMAP cohort, D) male AD network modules *versus* female network in the PFC of the ROSMAP cohort.

**Additional file 10**: **Supplemental Table 10.** The top network drivers of female AD. Candidate genes of the top AD-associated modules in the gene network of the PHG from the female AD subjects in the MSBB cohort and in the gene network of the PFC from the female AD subjects in the ROSMAP cohort. Genes that met any of three selection criteria were selected and further rank-ordered based on the strength of association with AD. The rank order was calculated based on multiple *p* values calculated from module-trait correlation and module-DEG enrichment analyses with a candidate score which ranges from 0 to 1. Number of the published literature on each specific candidate gene was also shown.

**Additional file 11**: **Supplemental Table 11.** The top network drivers of male AD. Candidate genes of the top AD-associated modules in the gene network of the PHG from the male AD subjects in the MSBB cohort and in the gene network of the PFC from the male AD subjects in the ROSMAP cohort. Genes that met any of three selection criteria were selected and further rank-ordered based on the strength of association with AD. The rank order was calculated based on multiple *p* values calculated from module-trait correlation and module-DEG enrichment analyses with a candidate score which ranges from 0 to 1. Number of the published literature on each specific candidate gene was also shown.

**Additional file 12**: **Supplemental Table 12.** Validation studies of top candidate gene LRP10 using postmortem human brain samples. The human brain samples from the PHG brain region of the MSBB cohort were used to perform qPCR and WB studies to determine if any statistically significant sex-specific differences in *lrp10* mRNA and LRP10 protein expression levels among groups of female and male AD and control subjects with different *APOE* genotypes.

**Additional file 13**: **Supplemental Table 13.** Cell type proportion in the mouse scRNA-seq data. The proportion of each brain cell type in total cell counts as well as the percentage of homeostasis-associated microglia* versus* damage-associated microglia in total microglial counts were shown in each group of female and male E3FAD and E4FAD control *versus* LRP10 OE mouse brains.

**Additional file 14**: **Supplemental Table 14.** Sub-clustering analysis of neurons in mouse scRNA-seq data. A) Number of cells of each neuronal subtype in each sample. B) Proportions of the four neuronal subtypes in each sample. C) Number of differentially expressed genesin each of neuronal subtypes induced by LRP10 OE. In each microglia subtype, differential expression was performed on four comparisons including Female LRP10 OE E4FAD *versus* Female E4FAD ctrl, Female LRP10 OE E3FAD *versus* Female E3FAD ctrl, Male LRP10 OE E4FAD *versus* Male E4FAD ctrl, and Male LRP10 OE E3FAD *versus* Male E3FAD ctrl.

**Additional file 15:** **Supplemental Table 15.** Number of differentially expressed genesin DAM and HAM induced by LRP10 Overexpression. In each microglia subtype, differential expression was performed on four comparisons including Female LRP10 OE E4FAD *versus* Female E4FAD ctrl, Female LRP10 OE E3FAD *versus* Female E3FAD ctrl, Male LRP10 OE E4FAD *versus* Male E4FAD ctrl, and Male LRP10 OE E3FAD *versus* Male E3FAD ctrl.

**Additional file 16**: **Supplemental Figure 1.** Differential Gene Expression Profiles of Female and Male AD *versus* Control. A) Multi-set intersection analysis of the DEG signatures from four sex specific comparisons including Male AD *versus* Male Control, Female AD *versus* Female Control, Female AD *versus* Male AD, and Female Control *versus* Male Control. The matrix of solid and empty circles at the bottom illustrates the “presence”or “absence”of the DEG sets in each intersection. The numbers to the right of the matrix are set sizes. The colored bars on the top of the matrix represent the overlap sizes with the color intensity showing *p* value. B) The PCA analysis of human samples including female AD, female control, male AD and male control. C) Left: Numbers of DEGs identified between AD *versus *control in each APOE genotype and sex group for the PHG brain region. Right: Numbers of DEGs identified between female *versus* male in each APOE genotype and disease group for the PHG brain region.

**Additional file 17**: **Supplemental Figure 2.** Venn diagrams of the DEG signatures identified between AD *versus* control in each sex in the PHG and the PFC. A) Venn diagrams of the DEG signatures identified between AD *versus* control with all APOE genotypes combined in each sex. In PHG, 343 up-regulated DEGs between AD *versus* control were shared between females and males, whereas 433 down-regulated DEGs between AD *versus* control were shared in both sex groups. Only 1 DEG was up-regulated in male AD when compared to male control group while down-regulated in female AD. On the other hand, very few DEGs were shared between these groups in the PFC region of the ROSMAP cohort. B) Venn diagrams of the DEG signatures identified between AD *versus* control in the PHG and PFC regions. For the up-regulated DEGs between AD *versus* control, 190 and 3,655 DEGs were specific to males and females in the PHG, respectively, whereas 1 and 22 DEGs were specific to males and females in the PFC, respectively. For the down-regulated DEGs, 132 and 2,336 DEGs were specific to males and females in the PHG, respectively, whereas 18 and 125 DEGs were specific to males and females in the PFC, respectively.

**Additional file 18**: **Supplemental Figure 3.** Differential Trend Analysis of DEGs between Female *versus* Male. A) Numbers of differentially trended genesbetween female *versus *male identified by Jonckheere and Spline trend analyses in four AD clinical traits, Braak stage, CERAD, and plaque density) and APOE genotype. BBSCORE or Braak score refers to Braak stage. B) ~ D) Numbers of overlapping DTGs between female *versus *male identified by Jonckheere analysis in B) APOE genotype, C) Braak stage and D) CERAD scores. E) Top DTGs showing significantly opposite expression trends between female *versus *male in each AD clinical trait.

**Additional file 19**: **Supplemental Figure 4.** Multi-set intersection analysis of the DTG signatures with respect to various clinical/pathological traits in the PHG of the MSBB cohort. The matrix of solid and empty circles at the bottom illustrated the “presence”or “absence”of the DEG sets in each intersection. The numbers to the right of the matrix were set sizes. The colored bars on the top of the matrix represented the overlap sizes with the color intensity showing *p *value.

**Additional file 20**: **Supplemental Figure 5.** Sex-Specific gene co-expression networks and gene modules. A) The global MEGENA network in the PHG from the female or maleAD subjects in the MSBB cohort. The modules at one particular compact scale were represented by different colors. B) The gene modules that were most enriched for neuronal and microglial marker genes in the male AD gene networks of the PHG in the MSBB cohort.

**Additional file 21**: **Supplemental Figure 6.** Enrichment of functional pathways in co-expressed gene modules. A) Significantly enriched pathways for the top modules in the female AD networks of the top modules in the sex-specific AD gene networks in the PHG of the MSBB cohort and the PFC in the ROSMAP cohort. B) Significantly enriched pathways for the top modules in the male AD networks in the PHG of the MSBB cohort and the PFC of the ROSMAP cohort. In the PHG region, the most enriched functional pathways across both female and male AD networks were oxidative phosphorylation and neurodegenerative disease pathways, such as Alzheimer’s disease pathway, Parkinson's disease pathway and Huntington’s disease pathway. The most enriched GO term pathways were enriched in immune system process and nervous system development.

**Additional file 22**: **Supplemental Figure 7.** LDLR-related protein 10 identified and Validated as a sex-specific key regulator of AD. A) Levels of *lrp10* mRNA by qPCR analysis were compared between AD *versus* control, male *versus* female in the PHG human brain samples. *N*=10-21/group, ANOVA with post-hoc tests to determine group differences for multiple comparisons and independent-samples *t*-tests for paired comparisons with *p<0.05. B) Levels of LRP10 protein by western blot analysis were compared between AD *versus* control, male *versus* female in the PHG human brain samples. *N*=10-20/group, ANOVA with post-hoc tests to determine group differences for multiple comparisons and independent-samples *t*-tests for paired comparisons with **p<0.01. C) Levels of LRP10 protein by western blot analysis were examined in hippocampal brain regions of 6-month-old female and male APOE3 and APOE4 as well as E3FAD and E4FAD. Left panel: 8 groups for comparison with breakdown by APOE genotypes; Right panel: 4 groups for comparison. *N*=4-8/group, ANOVA with post-hoc tests to determine group differences for multiple comparisons and independent-samples *t*-tests for paired comparisons with *p<0.05 ***p<0.001 ****p<0.0001. D) The specificity of the LRP10 antibody used in our study was confirmed by western blot analysis of samples with LRP10 OE or siRNA knockdown treatments. A representative western blot image of LRP10 and action was shown.

**Additional file 23**: **Supplemental Figure 8.** Characterization of AD-related phenotypes in female EFAD mice with LRP10 over-expression. A) ~ C) Levels of LRP10 protein, pTau, total Tau, oligomer and soluble Aβ_42_, soluble Aβ_40_ and APOE in mouse hippocampus of female E4FAD scramble control* versus* LRP10 OE. *N*=7-16/group; *p<0.05 **p<0.01 with unpaired T-tests with Welch’s corrections. Levels determined by western blot were presented as % of control, and levels determined by ELISA were presented as pg/ml equivalent to pg per 1mg of total proteins. D) Total numbers of IBA1^+^ microglia in the hippocampal regions were compared between scramble control* versus* LRP10 OE female E4FAD mice. Data were presented as % of controls. E) Levels of TNFα and IL-17 were determined by ELISA and data presented as pg/ml equivalent to pg per 1mg of total proteins. *p<0.05 by unpaired T-tests with Welch’s corrections.

**Additional file 24**: **Supplemental Figure 9.** Characterization of AD-related phenotypes in male EFAD mice with LRP10 over-expression. Levels of A) IL6 and IL10, B) pTau and total Tau, C) oligomer and soluble Aβ_42_, as well as soluble Aβ_40_ in mouse hippocampus of male E4FAD scramble control* versus* LRP10 OE. *N*=7-16/group; *p<0.05 with unpaired T-tests with Welch’s corrections. Levels determined by western blot were presented as % of control, and levels determined by ELISA were presented as pg/ml equivalent to pg per 1mg of total proteins. D) Quantification of amyloid plaque burden in E4FAD male mouse hippocampus by density was measured by size of all plaques in the brains of scramble *versus* LRP10 OE male E4FAD mice. Distribution of plaques measured by numbers of plaques in different sizes was compared between scramble *versus* LRP10 OE male E4FAD mouse brains as well.

**Additional file 25**: **Supplemental Figure 10.** Cell-type specific gene expression changes in LRP10 OE mouse brains. A) Volcano plots of RNA counts, mitochondrial and ribosome proportions ad well as B) scatter plots of RNA counts of all datasets from 8 experimental groups after quality control processes to remove cells with less than 200 genes or genes expressed less than 3 cells, or cells with mitochondrial proportion greater than 20% or ribosome proportion less than 5%, or cells with abnormally high RNA counts based on scatter plots.

**Additional file 26**: **Supplemental Figure 11.** Cell-type specific Changes in LRP10 OE mouse brains. A) UMAP visualization showing clustering of integrated dataset including all 8 experimental groups. B) UMAP visualization showing clustering of different brain cell types of each experimental group. C) UMAP visualization showing clustering of microglial subtypes of integrated dataset including all 8 experimental groups. D) UMAP visualization showing microglial subclustersof each experimental group.

**Additional file 27:** **Supplemental Figure 12.** Sub-clustering analysis of neurons in LRP10 OE mouse brains. A) UMAP visualization of neuronal subtypes from the sub-clustering analysis of all neurons. B) Proportions of neuronal subtypes in each experimental group. C) Multi-set intersection analysis of the sex and ApoE4 specific gene signatures in the neuron subtype from the comparisons including Female LRP10 OE E4FAD *versus* Female E4FAD ctrl and Male LRP10 OE E4FAD *versus* Male E4FAD ctrl. In each signature, the up- and down-regulated DEGs were separated for the intersection analysis. The matrix of solid and empty circles at the bottom illustrated the “presence”or “absence”of the DEG sets in each intersection. The numbers to the right of the matrix were set sizes. The colored bars on the top of the matrix represented the overlap sizes with the color intensity showing *p *value.

**Additional file 28**: **Supplemental Figure 13.** Enrichment of functional pathways in the cell type, sex and ApoE-genotype specific gene signatures induced by LRP10 OE.

**Additional file 29**: **Supplemental Figure 14.** Multi-set intersection analysis of the sex and ApoE-genotype specific gene signatures in in DAM and HAM induced by LRP10 OE. The matrix of solid and empty circles at the bottom illustrated the “presence”or “absence”of the DEG sets in each intersection. The numbers to the right of the matrix were set sizes. The colored bars on the top of the matrix represented the overlap sizes with the color intensity showing *p *value.

**Additional file 30:** **Supplemental Figure 15.** LRP10 binding partners. A) The positive hits that were validated by β-galactosidase assays. B) The brain cell type specific expression patterns of LRP10 and its binding partners CD34, NBR1 and ACBD3 in female E4FAD mouse brains of scramble control* versus* LRP10 OE. C) The expression patterns of LRP10 and its binding partners CD34, NBR1 and ACBD3 in microglial subclusters such as damage-associated microglia and homeostasis-associated microglia in female E4FAD mouse brains of scramble control* versus* LRP10 OE. D) The levels of LRP1, LDLR, LRP6 and LRP3 protein were examined in female E4FAD mouse brains of scramble control* versus* LRP10 OE. N=3-9/group; *p<0.05 by unpaired T-tests with Welch’s corrections.

## Data Availability

The datasets downloaded, generated and/or analyzed during the current study include the dataset from the MSBB study (adknowledgeprotal.synapse.org/syn319438), the dataset from the ROSMAP study (adknowledgeprotal.synapse.org/syn3219045) and the scRNA-seq dataset we generated and analyzed (synapse.org/syn29651620).

## Abbreviations

AAV: Adeno-associated virus
Aβ: Amyloid beta
AD: Alzheimer’s disease
ANOVA: Analysis of variances
APOE: Apolipoprotein E
*apoJ*: *apolipoprotein J*
APP: Amyloid precursor protein
BH: Benjamini–Hochberg’s
CDR: Clinical dementia rating
CERAD: Consortium to establish a registry for alzheimer’s disease
ChEl: Cholinesterase inhibitor
CPM: Count per million
DAM: Damage-associated microglia
DE: Differential expression
DEGs: Differentially expressed genes
DTGs: Differentially trended genes
ELISA: Enzyme-linked immunosorbent assay
FC: Fold change
FDR: False discovery rate
FPKM: Fragments per kilobase million
GAPDH: Glyceraldehyde-3-phosphate dehydrogenase
GFAP: Glial fibrillary acidic protein
GO: Gene ontology
GOC: Gain of connectivity
GWAS: Genome-wide association studies
HAM: Homeostasis-associated microglia
IACUC: Institutional animal care and use committees
IBA-1: Ionized calcium binding adaptor molecule 1
IGAP: International genomics of Alzheimer’s project
IL6: Interleukin 6
IL10: Interleukin 10
ISMMS: Icahn School of Medicine at Mount Sinai
JJPVAMC: James J. Peters VA medical center
KD: Knock-down
KDA: Key driver analysis
KEGG: Kyoto encyclopedia of genes and genomes
KI: Knock-in
LDLR: Low-density lipoprotein receptor
LOC: Loss of connectivity
*lrp10*: Lipoprotein receptor related protein 10
MDC: Modular differential connectivity
MEGENA: Multiscale embedded gene co-expression network analysis
MSBB: Mount Sinai brain bank
NIH: National institutes of health
NOR: Novel object recognition
OE: Over-expression
PCA: Principal component analysis
PFC: Prefrontal cortex
PHG: Para-hippocampal gyrus
PMI: Postmortem interval
pTau: Phosphorylated-Tau
QC: Quality control
qPCR: Quantitative polymerase chain reaction
RIN: RNA integrity number
ROS: Reactive oxidative species
ROSMAP: Religious orders study and Rush memory and aging project
scRNA-seq: Single cell RNA sequencing
SDS-PAGE: Sodium dodecyl sulphate–polyacrylamide gel electrophoresis
shRNA: Short hairpin RNA
TCGs: Trait-associated genes
TGN: Trans-Golgi network
TMM: Trimmed mean of the M-values
TNFα: Tumor necrosis factor alpha
UMAP: Uniform manifold approximation and projection
UMIs: Unique molecular identifiers
