# Supplementary material for: Sex specific molecular networks and key drivers of Alzheimer’s disease
Source: Mol Neurodegener. 2023 Jun 20;18:39. doi: 10.1186/s13024-023-00624-5 (PMC10280841; doi:10.1186/s13024-023-00624-5)
Supplement: Supplementary file 12 — Additional file 12: Supplemental Table 12. Validation studies of top candidate gene LRP10 using postmortem human brain samples. The human brain samples from the PHG brain region of the MSBB cohort were used to perform qPCR and WB studies to determine if any statistically significant sex-specific differences in lrp10 mRNA and LRP10 protein expression levels among groups of female and male AD and control subjects with different APOE genotypes. [file 13024_2023_624_MOESM12_ESM.docx]

**Supplemental Table 11. Comparison of LRP10 expression levels among different groups.** The human brain samples from the PHG brain region of the MSBB cohort were used to perform qPCR (top panels) and WB (bottom panels) studies to determine if any statistically significant sex-specific differences in *lrp10* mRNA and LRP10 protein expression levels among groups: female *versus* male, AD *versus* control subjects with different *APOE* genotypes (*p* values<0.05 considered as statistically significant).

| Log_2_ FC | *APOE* 3/3  F AD *vs* M AD | *APOE* 3/4  F AD *vs* M AD | *APOE* 3/3  F ctrl *vs* M ctrl | *APOE* 3/4  F ctrl *vs* M ctrl | *APOE* 3/3  F AD *vs* F ctrl | *APOE* 3/4  F AD *vs* F ctrl |
| --- | --- | --- | --- | --- | --- | --- |
| *lrp10* mRNA | -0.340  *p*=0.71 | **-0.885**  ***p*=0.02** | 0.519  *p*=0.466 | -1.716  *p*=0.39 | 0.078  *p*=0.92 | **2.042**  ***p<*0.0001** |
| Log_2_ FC | *APOE* 3/3  M AD *vs* M ctrl | *APOE* 3/4  M AD *vs* M ctrl | F AD *vs* M AD | F ctrl *vs* M ctrl | F AD *vs* F ctrl | M AD *vs* M ctrl |
| *lrp10* mRNA | 0.938  *p*=0.19 | 0.262  *p*=0.71 | -0.613  *p*=0.17 | -0.360  *p*=0.57 | 0.840  *p*=0.17 | 0.779  *p*=0.17 |

| Log_2_ FC | *APOE* 3/3  F AD *vs* M AD | *APOE* 3/4  F AD *vs* M AD | *APOE* 3/3  F ctrl *vs* M ctrl | *APOE* 3/4  F ctrl *vs* M ctrl | *APOE* 3/3  F AD *vs* F ctrl | *APOE* 3/4  F AD *vs* F ctrl |
| --- | --- | --- | --- | --- | --- | --- |
| LRP10 protein | 0.144  *p*=0.36 | **0.535**  ***p*=0.02** | -0.084  *p*=0.73 | 0.398  *p*=0.13 | 0.018  *p*=0.91 | **-0.990**  ***p*=0.001** |
| Log_2_ FC | *APOE* 3/3  M AD *vs* M ctrl | *APOE* 3/4  M AD *vs* M ctrl | F AD *vs* M AD | F ctrl *vs* M ctrl | F AD *vs* F ctrl | M AD *vs* M ctrl |
| LRP10 protein | -0.209  *p*=0.24 | **-1.127**  ***p<*0.0001** | 0.299  *p*=0.09 | 0.08  *p*=0.58 | **-0.466**  ***p*=0.03** | **-0.6850**  ***p*=0.005** |

| Cell type | E3FAD  F ctrl | E3FAD  F LRP10 OE | E4FAD  F ctrl | E4FAD  F LRP10 OE | E3FAD  M ctrl | E3FAD  M LRP10 OE | E4FAD  M ctrl | E4FAD  M LRP10 OE |
| --- | --- | --- | --- | --- | --- | --- | --- | --- |
| microglia | 35.32 | 39.67 | 50.25 | **39.94** | 39.75 | 31.97 | 38.86 | **56.83** |
| oligodendrocyte | 14.53 | 10.98 | 4.12 | **7.26** | 13.32 | 9.5 | 11.45 | **8.75** |
| astrocyte | 27.58 | 20.39 | 28.7 | **30.91** | 20.65 | 22.35 | 28.48 | **15.96** |
| neuron | 13.48 | 20.36 | 10.54 | **14.79** | 14.95 | **24.38** | 10.89 | **8.43** |
| OPC | 2.1 | 1.44 | 0.83 | 1.2 | 2 | 1.6 | 1.48 | 1.19 |
| endothelia | 6.98 | 7.15 | 5.56 | 5.9 | 10.23 | 10.2 | 8.84 | 8.43 |
| Microglial subtype | E3FAD  F ctrl | E3FAD  F LRP10 OE | E4FAD  F ctrl | E4FAD  F LRP10 OE | E3FAD  M ctrl | E3FAD  M LRP10 OE | E4FAD  M ctrl | E4FAD  M LRP10 OE |
| homeostatic | 70.05 | 68.25 | 69.63 | **66.17** | 74.14 | 73.45 | 69.24 | **73.57** |
| DAM | 29.95 | 31.75 | 30.37 | **33.83** | 25.86 | 26.55 | 30.76 | **26.43** |

**Supplemental Table 5. Cell Percentage in sc-RNA-seq Analysis.** The proportion of each brain cell type in total cell counts as well as percentage of homeostatic vs damage-associated microglia (DAM) in total microglial counts are shown in each group of female and male E3FAD and E4FAD control *versus* LRP10 OE mouse brains.
