# Supplementary material for: Sex specific molecular networks and key drivers of Alzheimer’s disease
Source: Mol Neurodegener. 2023 Jun 20;18:39. doi: 10.1186/s13024-023-00624-5 (PMC10280841; doi:10.1186/s13024-023-00624-5)
Supplement: Supplementary file 13 — Additional file 13: Supplemental Table 13. Cell type proportion in the mouse scRNA-seq data. The proportion of each brain cell type in total cell counts as well as the percentage of homeostasis-associated microglia versus damage-associated microglia in total microglial counts were shown in each group of female and male E3FAD and E4FAD control versus LRP10 OE mouse brains. [file 13024_2023_624_MOESM13_ESM.docx]

**Supplemental Table 13. Cell Percentage in sc-RNA-seq Analysis.** The proportion of each brain cell type in total cell counts as well as percentage of homeostatic vs damage-associated microglia (DAM) in total microglial counts are shown in each group of female and male E3FAD and E4FAD control *versus* LRP10 OE mouse brains.

| Cell type | E3FAD  F ctrl | E3FAD  F LRP10 OE | E4FAD  F ctrl | E4FAD  F LRP10 OE | E3FAD  M ctrl | E3FAD  M LRP10 OE | E4FAD  M ctrl | E4FAD  M LRP10 OE |
| --- | --- | --- | --- | --- | --- | --- | --- | --- |
| microglia | 35.32 | 39.67 | 50.25 | **39.94** | 39.75 | 31.97 | 38.86 | **56.83** |
| oligodendrocyte | 14.53 | 10.98 | 4.12 | **7.26** | 13.32 | 9.5 | 11.45 | **8.75** |
| astrocyte | 27.58 | 20.39 | 28.7 | **30.91** | 20.65 | 22.35 | 28.48 | **15.96** |
| neuron | 13.48 | 20.36 | 10.54 | **14.79** | 14.95 | **24.38** | 10.89 | **8.43** |
| OPC | 2.1 | 1.44 | 0.83 | 1.2 | 2 | 1.6 | 1.48 | 1.19 |
| endothelia | 6.98 | 7.15 | 5.56 | 5.9 | 10.23 | 10.2 | 8.84 | 8.43 |
| Microglial subtype | E3FAD  F ctrl | E3FAD  F LRP10 OE | E4FAD  F ctrl | E4FAD  F LRP10 OE | E3FAD  M ctrl | E3FAD  M LRP10 OE | E4FAD  M ctrl | E4FAD  M LRP10 OE |
| homeostatic | 70.05 | 68.25 | 69.63 | **66.17** | 74.14 | 73.45 | 69.24 | **73.57** |
| DAM | 29.95 | 31.75 | 30.37 | **33.83** | 25.86 | 26.55 | 30.76 | **26.43** |
