# Supplementary figures and images for: Sex specific molecular networks and key drivers of Alzheimer’s disease
Source: Mol Neurodegener. 2023 Jun 20;18:39. doi: 10.1186/s13024-023-00624-5 (PMC10280841; doi:10.1186/s13024-023-00624-5)

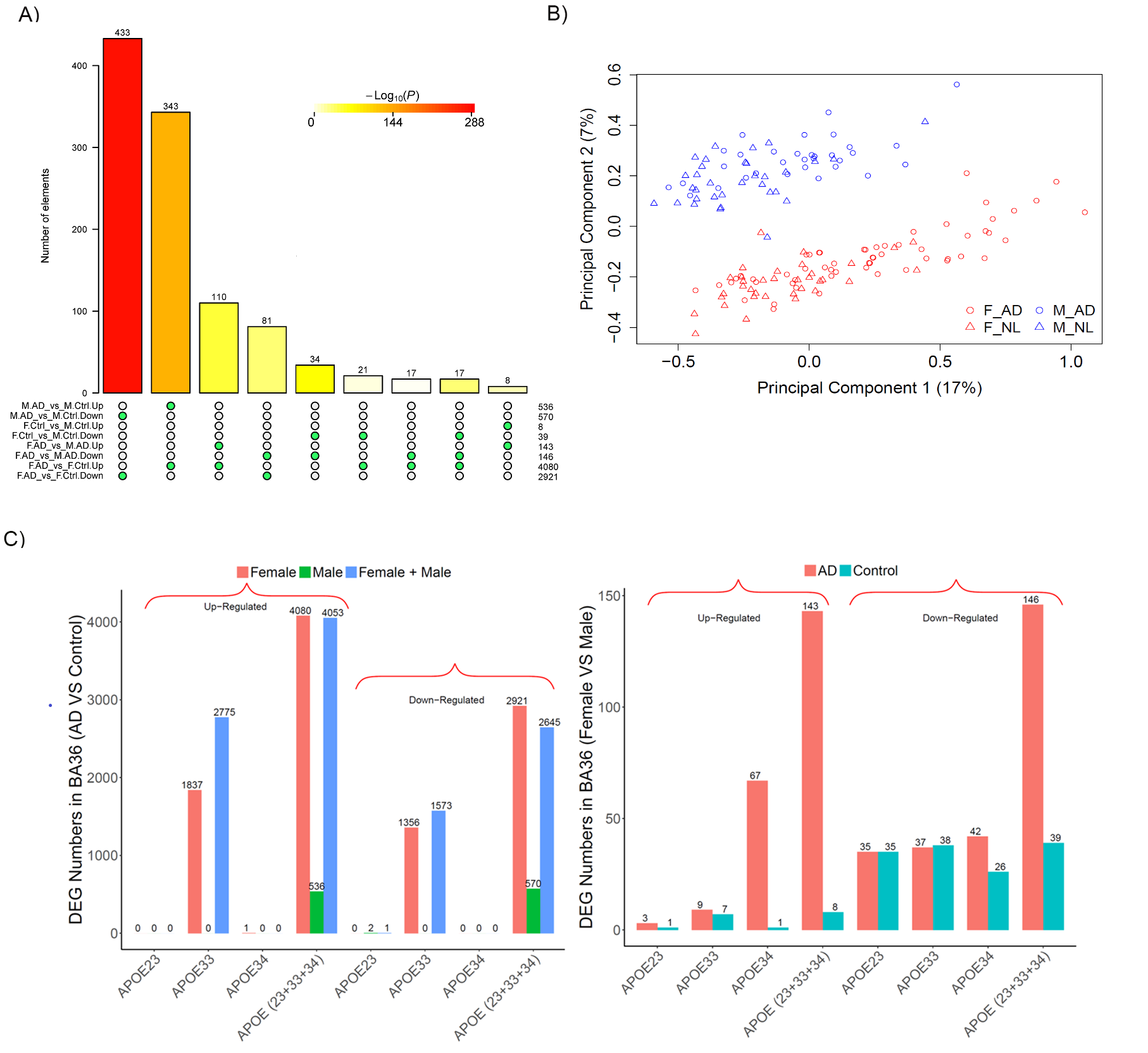

Supplement: Supplementary file 16 — Additional file 16: Supplemental Figure 1. Differential Gene Expression Profiles of Female and Male AD versus Control. A) Multi-set intersection analysis of the DEG signatures from four sex specific comparisons including Male AD versus Male Control, Female AD versus Female Control, Female AD versus Male AD, and Female Control versus Male Control. The matrix of solid and empty circles at the bottom illustrates the “presence”or “absence”of the DEG sets in each intersection. The numbers to the right of the matrix are set sizes. The colored bars on the top of the matrix represent the overlap sizes with the color intensity showing p value. B) The PCA analysis of human samples including female AD, female control, male AD and male control. C) Left: Numbers of DEGs identified between AD versus control in each APOE genotype and sex group for the PHG brain region. Right: Numbers of DEGs identified between female versus male in each APOE genotype and disease group for the PHG brain region. [file 13024_2023_624_MOESM16_ESM.tif]

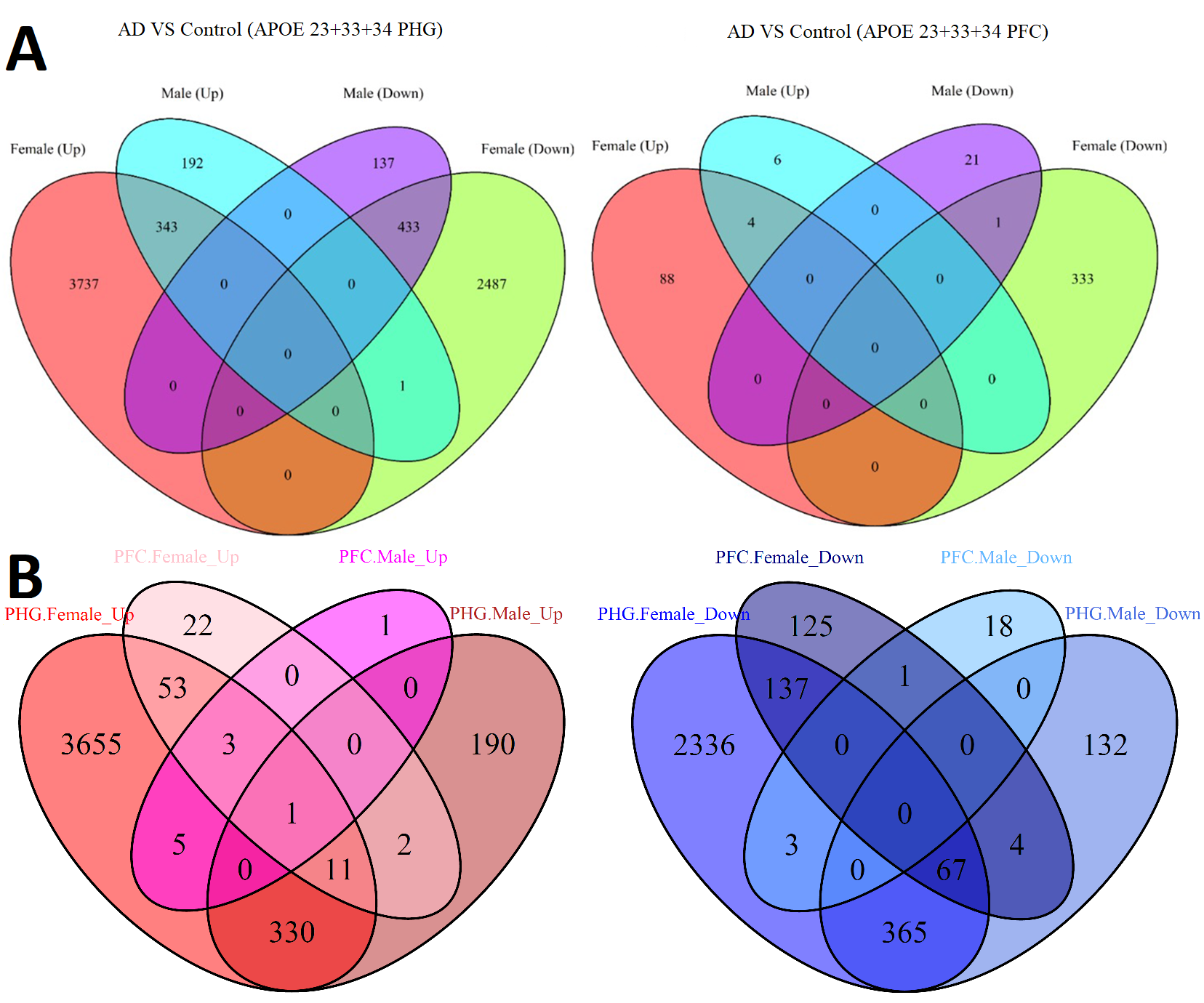

Supplement: Supplementary file 17 — Additional file 17: Supplemental Figure 2. Venn diagrams of the DEG signatures identified between AD versus control in each sex in the PHG and the PFC. A) Venn diagrams of the DEG signatures identified between AD versus control with all APOE genotypes combined in each sex. In PHG, 343 up-regulated DEGs between AD versus control were shared between females and males, whereas 433 down-regulated DEGs between AD versus control were shared in both sex groups. Only 1 DEG was up-regulated in male AD when compared to male control group while down-regulated in female AD. On the other hand, very few DEGs were shared between these groups in the PFC region of the ROSMAP cohort. B) Venn diagrams of the DEG signatures identified between AD versus control in the PHG and PFC regions. For the up-regulated DEGs between AD versus control, 190 and 3,655 DEGs were specific to males and females in the PHG, respectively, whereas 1 and 22 DEGs were specific to males and females in the PFC, respectively. For the down-regulated DEGs, 132 and 2,336 DEGs were specific to males and females in the PHG, respectively, whereas 18 and 125 DEGs were specific to males and females in the PFC, respectively. [file 13024_2023_624_MOESM17_ESM.tif]

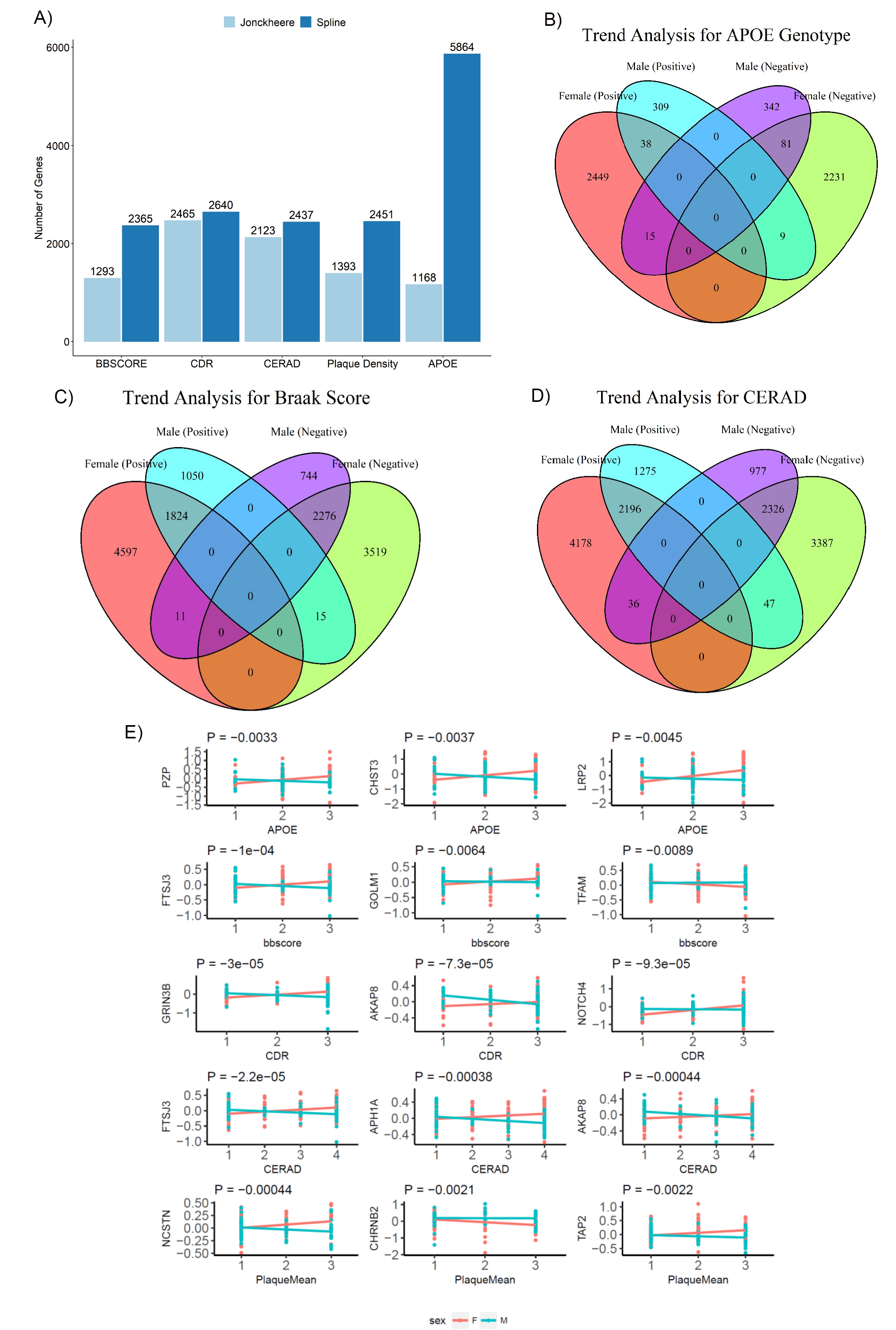

Supplement: Supplementary file 18 — Additional file 18: Supplemental Figure 3. Differential Trend Analysis of DEGs between Female versus Male. A) Numbers of differentially trended genesbetween female versus male identified by Jonckheere and Spline trend analyses in four AD clinical traits, Braak stage, CERAD, and plaque density) and APOE genotype. BBSCORE or Braak score refers to Braak stage. B) ~ D) Numbers of overlapping DTGs between female versus male identified by Jonckheere analysis in B) APOE genotype, C) Braak stage and D) CERAD scores. E) Top DTGs showing significantly opposite expression trends between female versus male in each AD clinical trait. [file 13024_2023_624_MOESM18_ESM.tif]

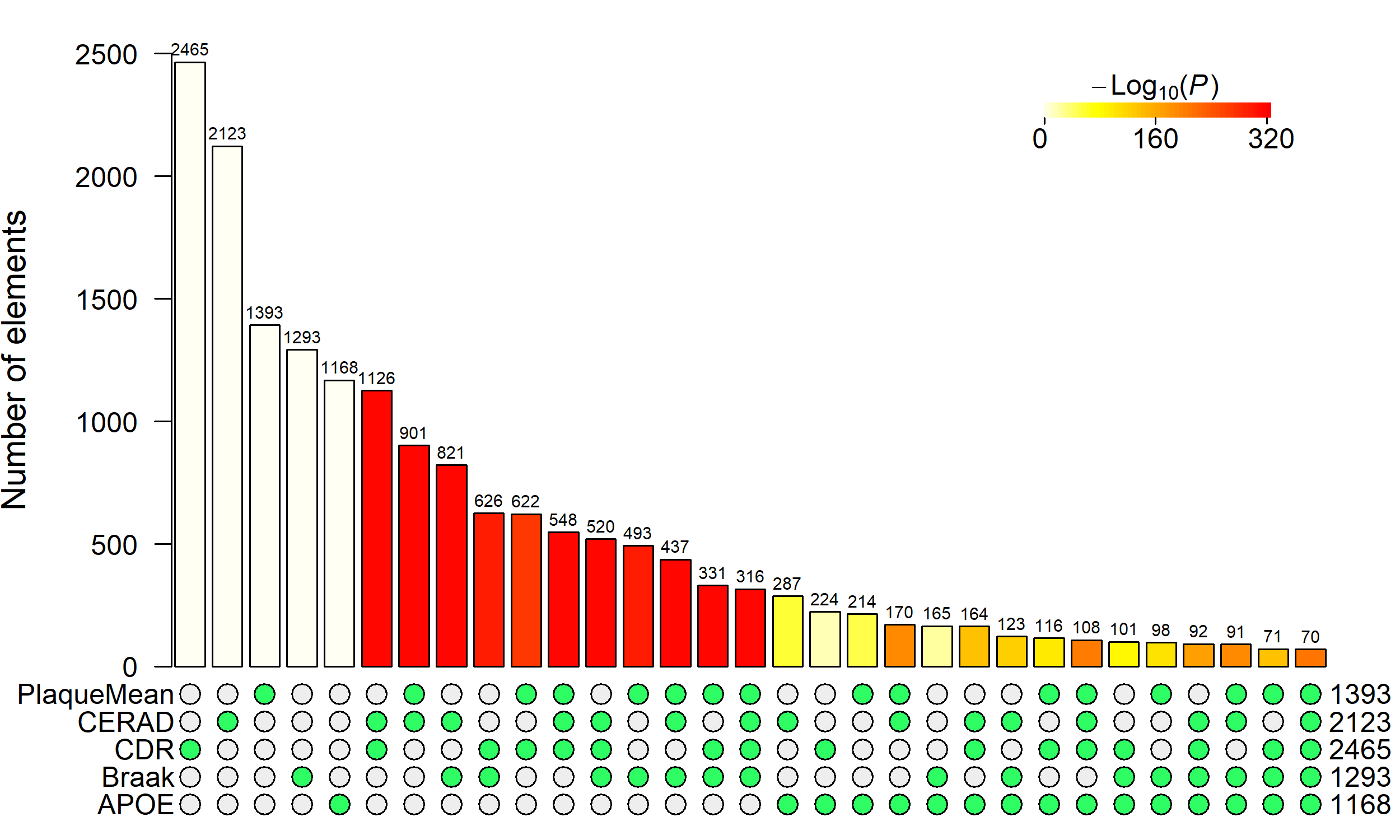

Supplement: Supplementary file 19 — Additional file 19: Supplemental Figure 4. Multi-set intersection analysis of the DTG signatures with respect to various clinical/pathological traits in the PHG of the MSBB cohort. The matrix of solid and empty circles at the bottom illustrated the “presence”or “absence”of the DEG sets in each intersection. The numbers to the right of the matrix were set sizes. The colored bars on the top of the matrix represented the overlap sizes with the color intensity showing p value. [file 13024_2023_624_MOESM19_ESM.tif]

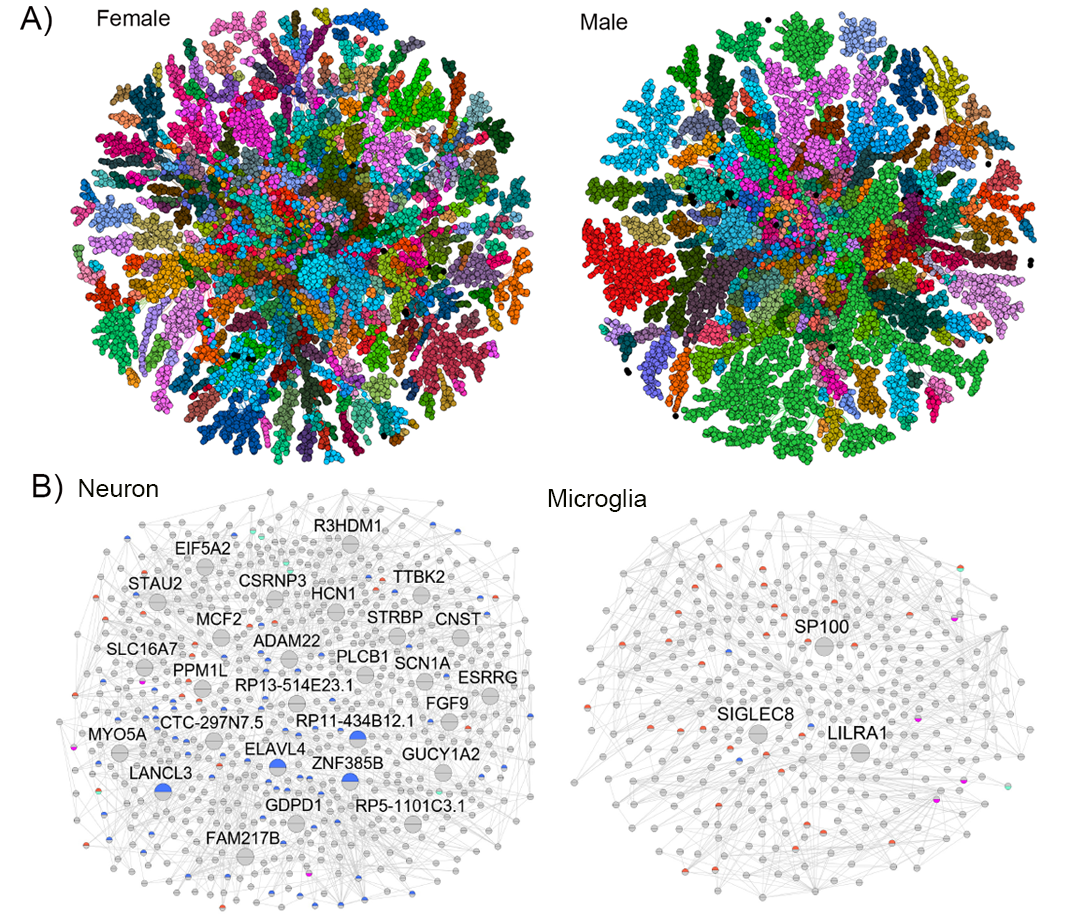

Supplement: Supplementary file 20 — Additional file 20: Supplemental Figure 5. Sex-Specific gene co-expression networks and gene modules. A) The global MEGENA network in the PHG from the female or maleAD subjects in the MSBB cohort. The modules at one particular compact scale were represented by different colors. B) The gene modules that were most enriched for neuronal and microglial marker genes in the male AD gene networks of the PHG in the MSBB cohort. [file 13024_2023_624_MOESM20_ESM.tif]

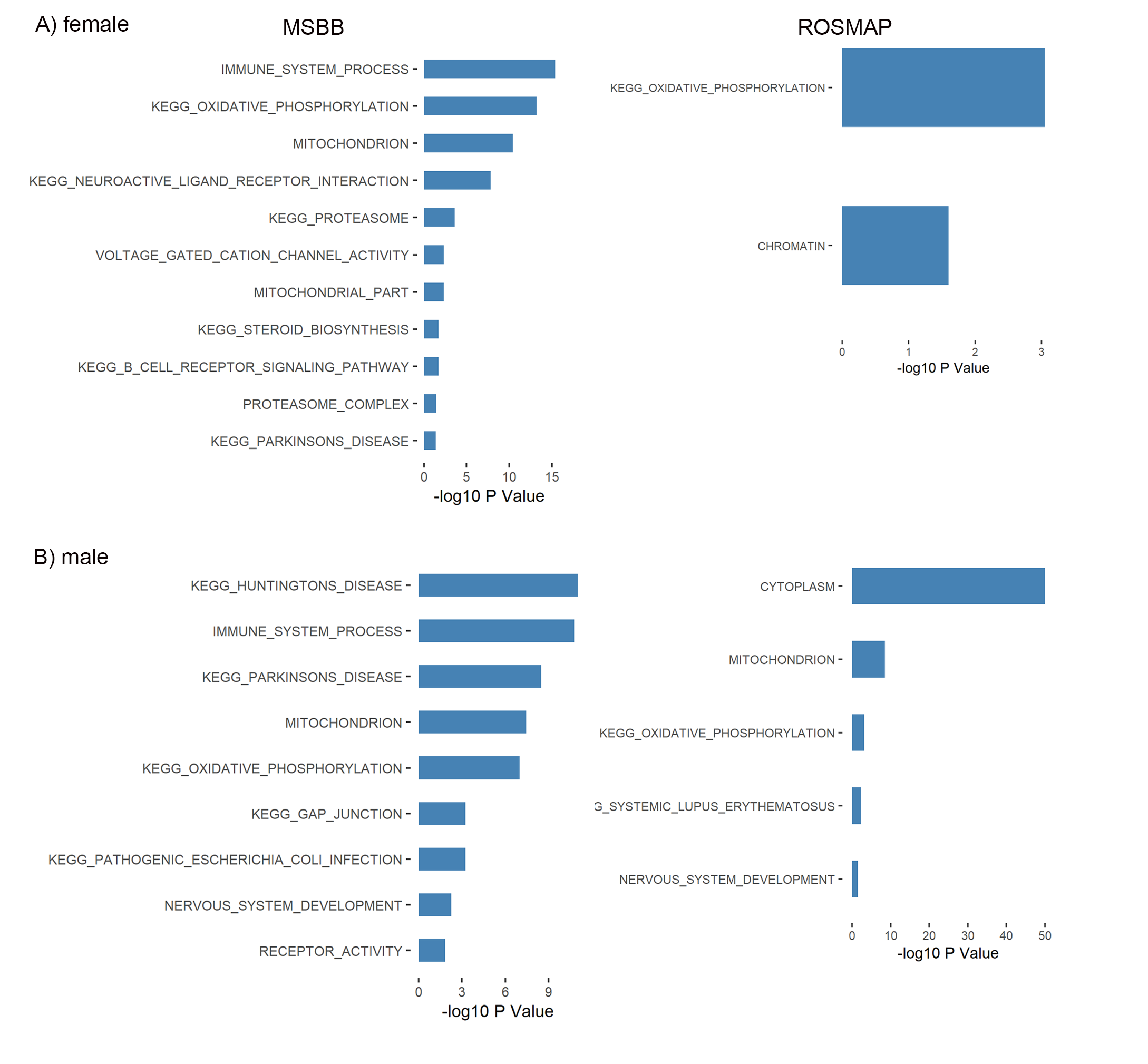

Supplement: Supplementary file 21 — Additional file 21: Supplemental Figure 6. Enrichment of functional pathways in co-expressed gene modules. A) Significantly enriched pathways for the top modules in the female AD networks of the top modules in the sex-specific AD gene networks in the PHG of the MSBB cohort and the PFC in the ROSMAP cohort. B) Significantly enriched pathways for the top modules in the male AD networks in the PHG of the MSBB cohort and the PFC of the ROSMAP cohort. In the PHG region, the most enriched functional pathways across both female and male AD networks were oxidative phosphorylation and neurodegenerative disease pathways, such as Alzheimer’s disease pathway, Parkinson's disease pathway and Huntington’s disease pathway. The most enriched GO term pathways were enriched in immune system process and nervous system development. [file 13024_2023_624_MOESM21_ESM.tif]

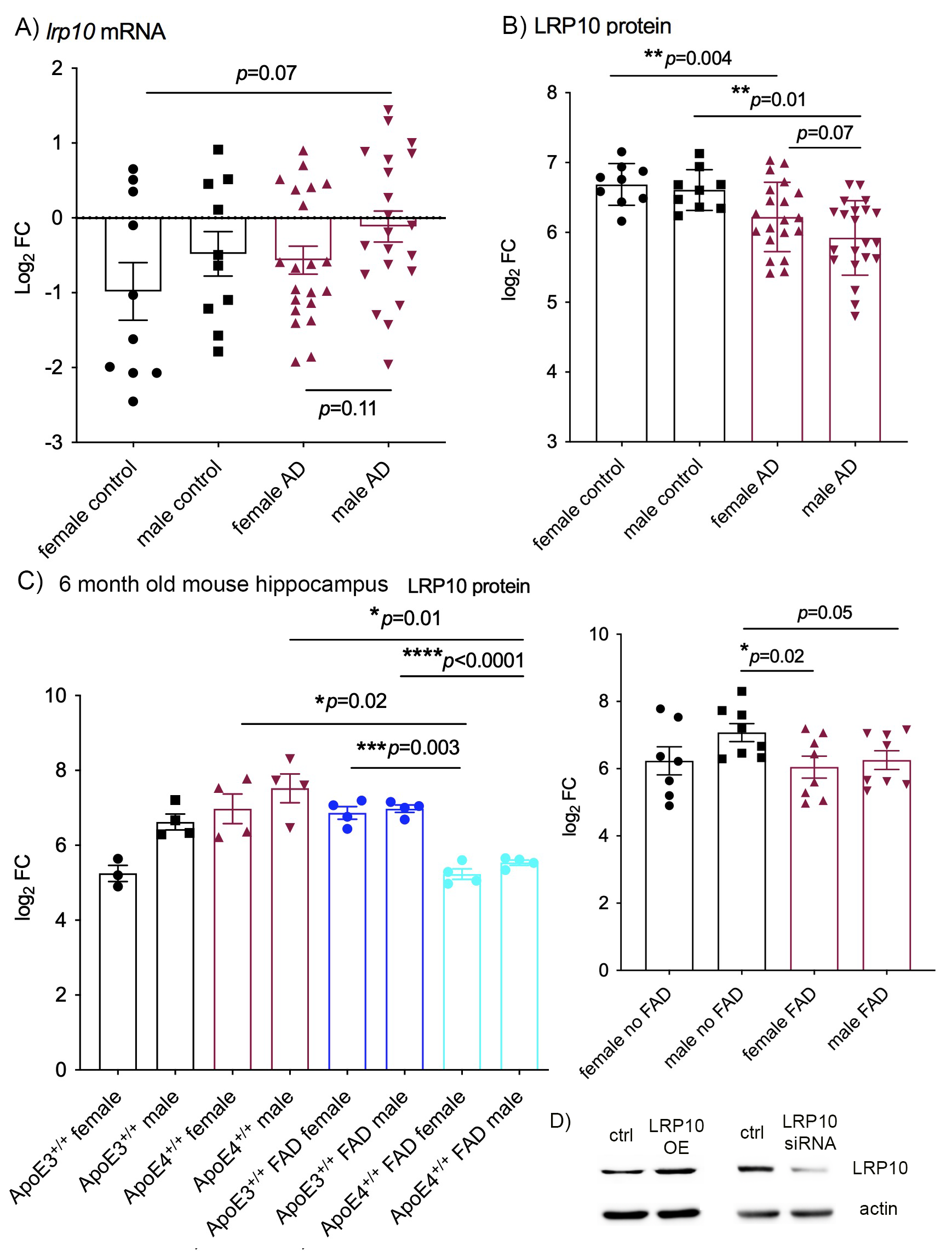

Supplement: Supplementary file 22 — Additional file 22: Supplemental Figure 7. LDLR-related protein 10 identified and Validated as a sex-specific key regulator of AD. A) Levels of lrp10 mRNA by qPCR analysis were compared between AD versus control, male versus female in the PHG human brain samples. N=10-21/group, ANOVA with post-hoc tests to determine group differences for multiple comparisons and independent-samples t-tests for paired comparisons with *p<0.05. B) Levels of LRP10 protein by western blot analysis were compared between AD versus control, male versus female in the PHG human brain samples. N=10-20/group, ANOVA with post-hoc tests to determine group differences for multiple comparisons and independent-samples t-tests for paired comparisons with **p<0.01. C) Levels of LRP10 protein by western blot analysis were examined in hippocampal brain regions of 6-month-old female and male APOE3 and APOE4 as well as E3FAD and E4FAD. Left panel: 8 groups for comparison with breakdown by APOE genotypes; Right panel: 4 groups for comparison. N=4-8/group, ANOVA with post-hoc tests to determine group differences for multiple comparisons and independent-samples t-tests for paired comparisons with *p<0.05 ***p<0.001 ****p<0.0001. D) The specificity of the LRP10 antibody used in our study was confirmed by western blot analysis of samples with LRP10 OE or siRNA knockdown treatments. A representative western blot image of LRP10 and action was shown. [file 13024_2023_624_MOESM22_ESM.tif]

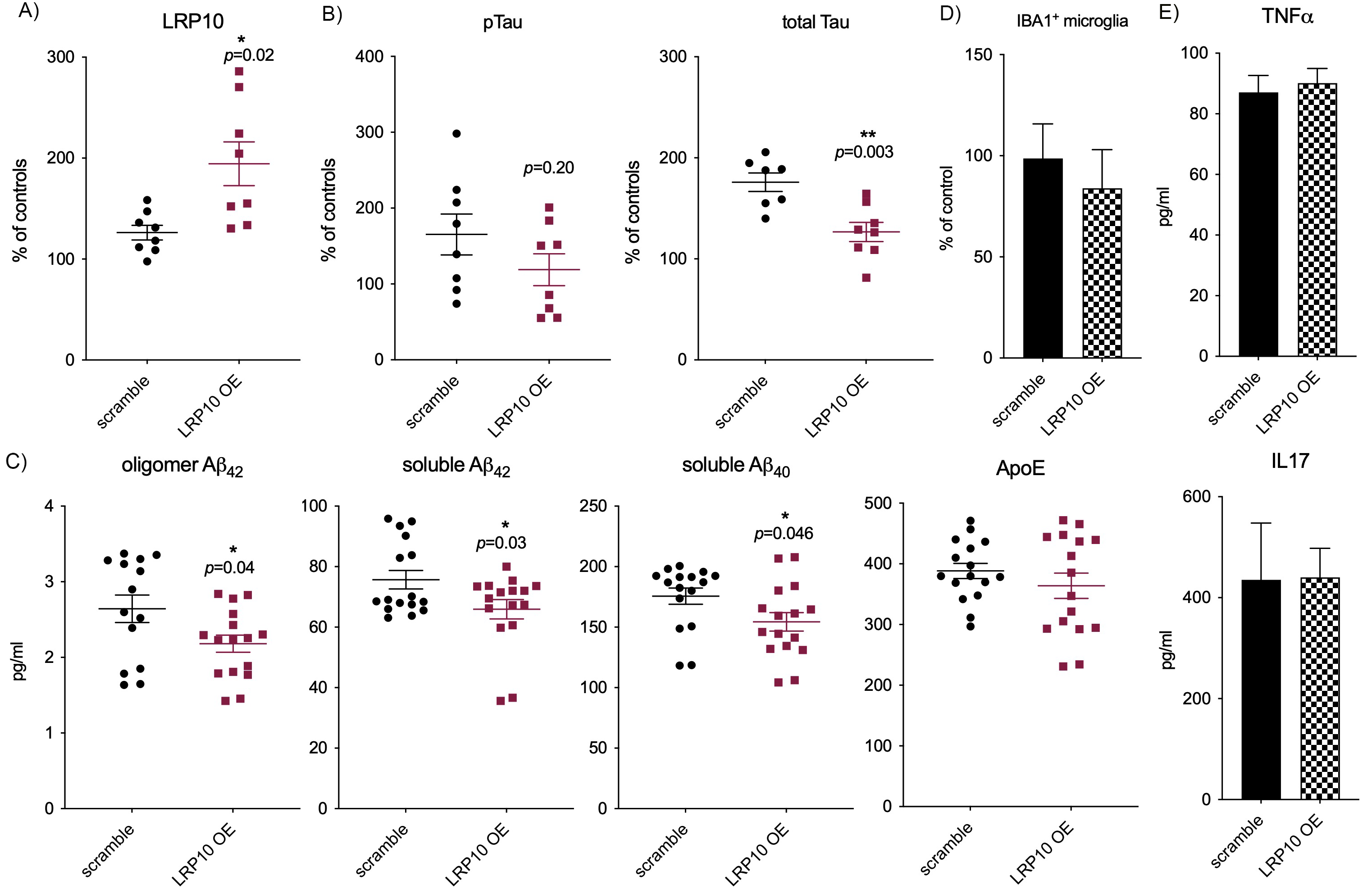

Supplement: Supplementary file 23 — Additional file 23: Supplemental Figure 8. Characterization of AD-related phenotypes in female EFAD mice with LRP10 over-expression. A) ~ C) Levels of LRP10 protein, pTau, total Tau, oligomer and soluble Aβ42, soluble Aβ40 and APOE in mouse hippocampus of female E4FAD scramble control versus LRP10 OE. N=7-16/group; *p<0.05 **p<0.01 with unpaired T-tests with Welch’s corrections. Levels determined by western blot were presented as % of control, and levels determined by ELISA were presented as pg/ml equivalent to pg per 1mg of total proteins. D) Total numbers of IBA1+ microglia in the hippocampal regions were compared between scramble control versus LRP10 OE female E4FAD mice. Data were presented as % of controls. E) Levels of TNFα and IL-17 were determined by ELISA and data presented as pg/ml equivalent to pg per 1mg of total proteins. *p<0.05 by unpaired T-tests with Welch’s corrections. [file 13024_2023_624_MOESM23_ESM.tif]

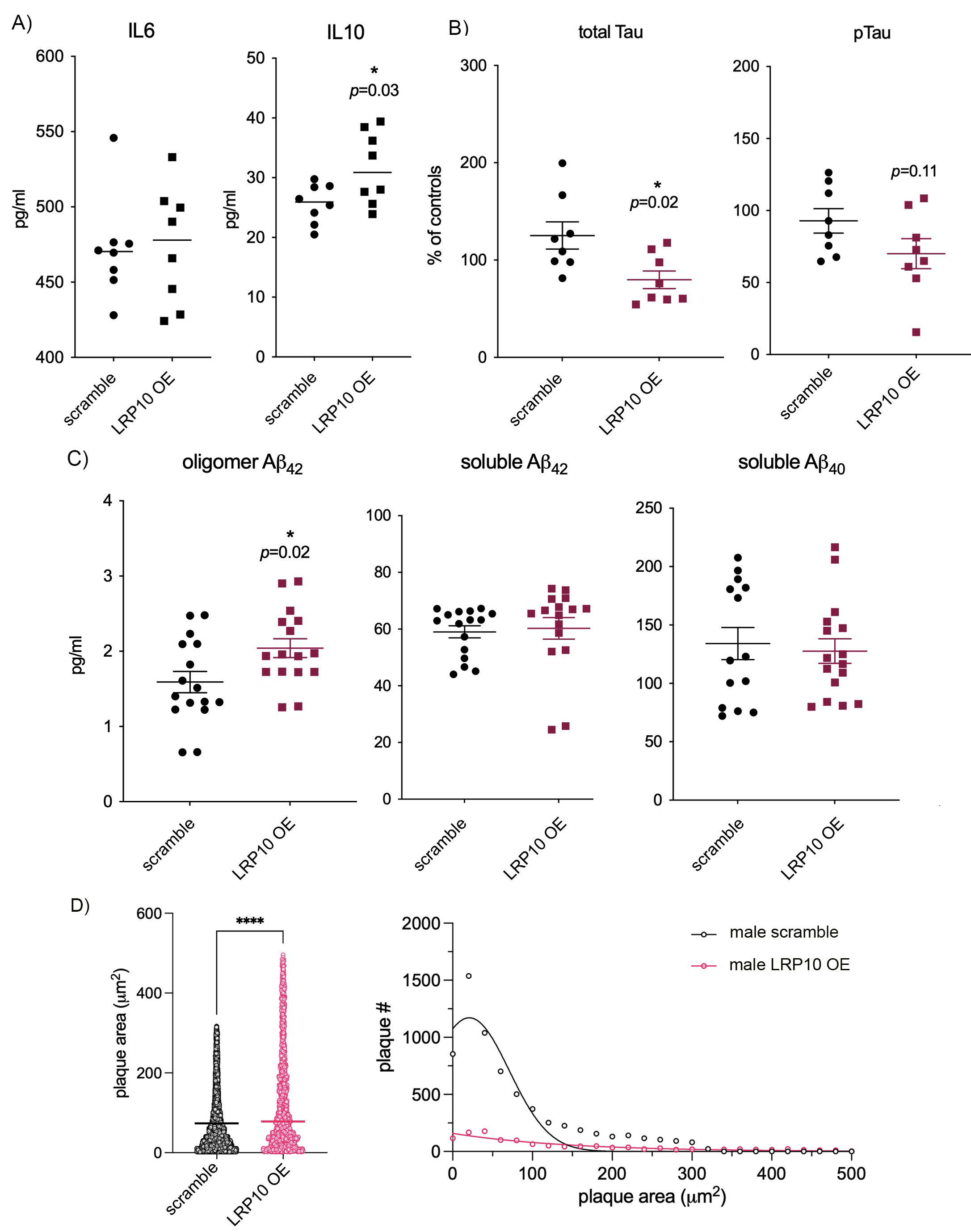

Supplement: Supplementary file 24 — Additional file 24: Supplemental Figure 9. Characterization of AD-related phenotypes in male EFAD mice with LRP10 over-expression. Levels of A) IL6 and IL10, B) pTau and total Tau, C) oligomer and soluble Aβ42, as well as soluble Aβ40 in mouse hippocampus of male E4FAD scramble control versus LRP10 OE. N=7-16/group; *p<0.05 with unpaired T-tests with Welch’s corrections. Levels determined by western blot were presented as % of control, and levels determined by ELISA were presented as pg/ml equivalent to pg per 1mg of total proteins. D) Quantification of amyloid plaque burden in E4FAD male mouse hippocampus by density was measured by size of all plaques in the brains of scramble versus LRP10 OE male E4FAD mice. Distribution of plaques measured by numbers of plaques in different sizes was compared between scramble versus LRP10 OE male E4FAD mouse brains as well. [file 13024_2023_624_MOESM24_ESM.tif]

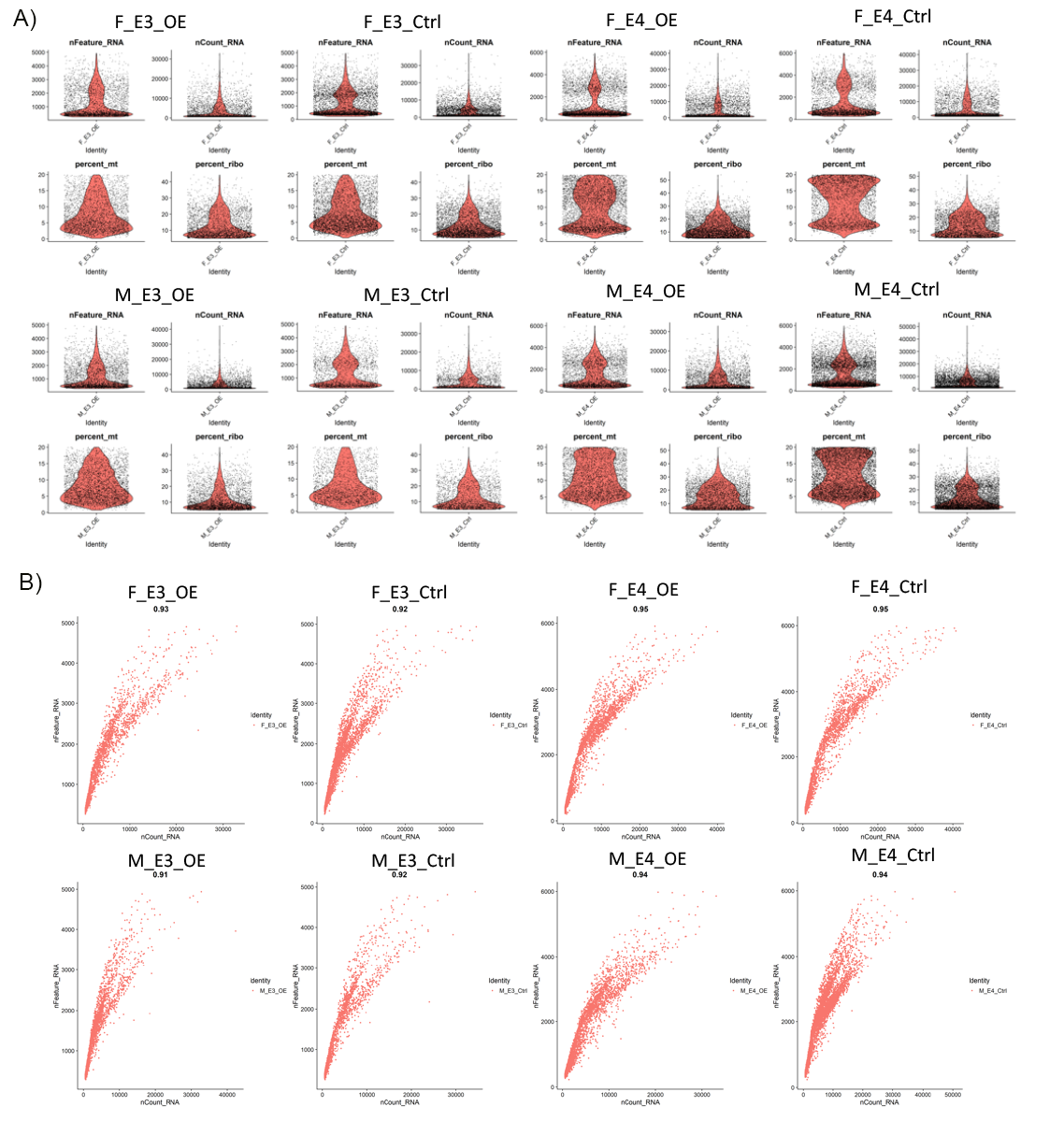

Supplement: Supplementary file 25 — Additional file 25: Supplemental Figure 10. Cell-type specific gene expression changes in LRP10 OE mouse brains. A) Volcano plots of RNA counts, mitochondrial and ribosome proportions ad well as B) scatter plots of RNA counts of all datasets from 8 experimental groups after quality control processes to remove cells with less than 200 genes or genes expressed less than 3 cells, or cells with mitochondrial proportion greater than 20% or ribosome proportion less than 5%, or cells with abnormally high RNA counts based on scatter plots. [file 13024_2023_624_MOESM25_ESM.tif]

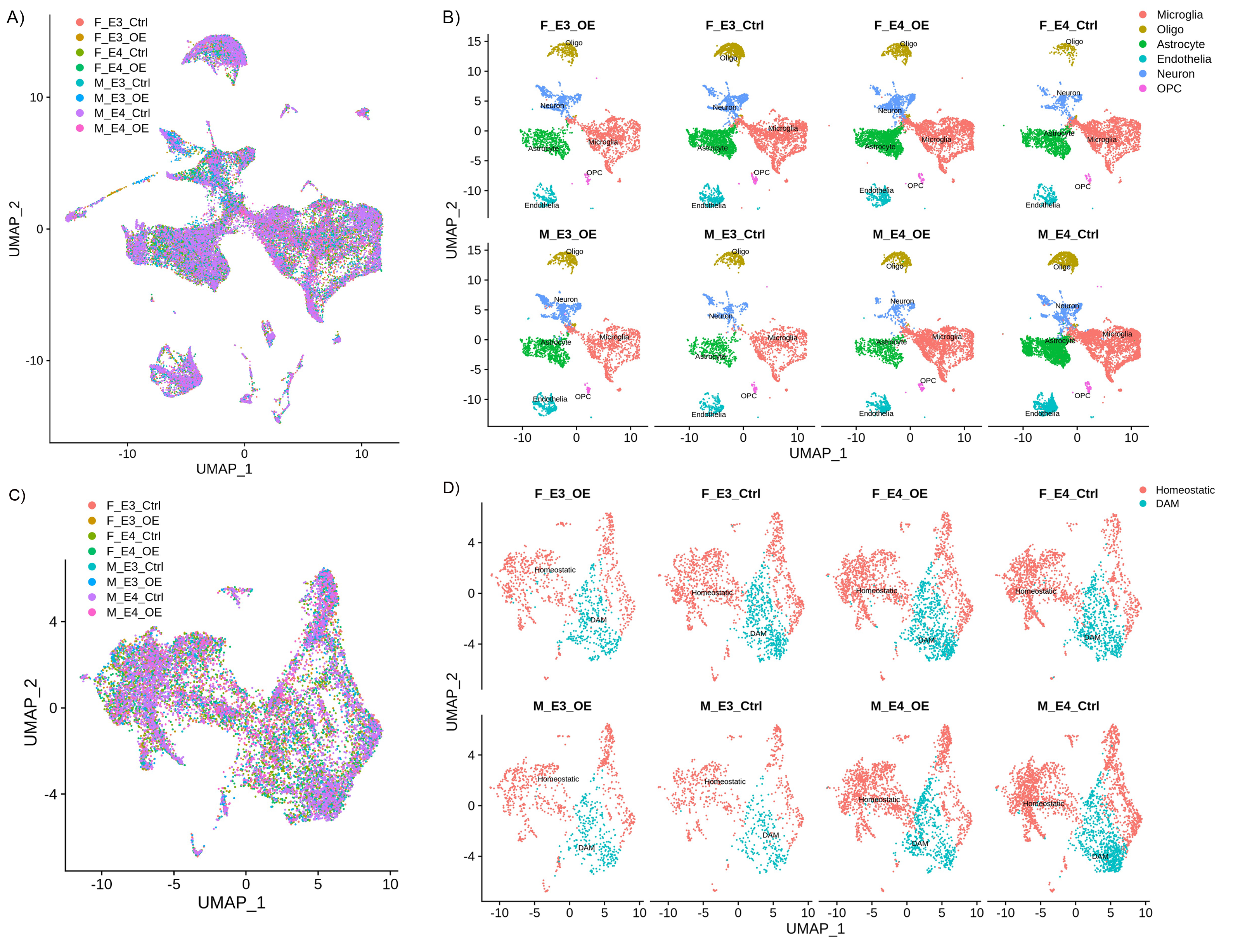

Supplement: Supplementary file 26 — Additional file 26: Supplemental Figure 11. Cell-type specific Changes in LRP10 OE mouse brains. A) UMAP visualization showing clustering of integrated dataset including all 8 experimental groups. B) UMAP visualization showing clustering of different brain cell types of each experimental group. C) UMAP visualization showing clustering of microglial subtypes of integrated dataset including all 8 experimental groups. D) UMAP visualization showing microglial subclustersof each experimental group. [file 13024_2023_624_MOESM26_ESM.tif]

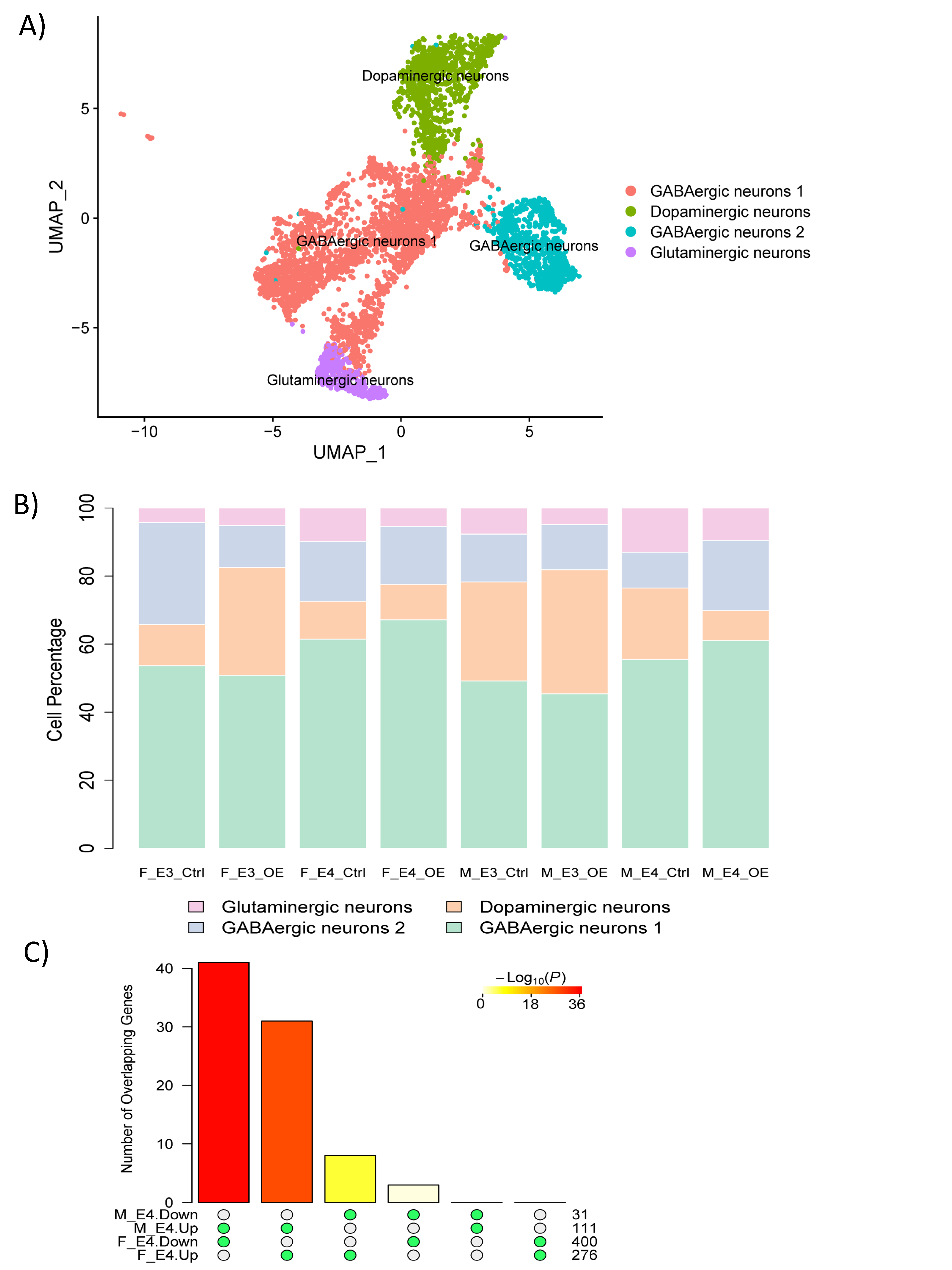

Supplement: Supplementary file 27 — Additional file 27: Supplemental Figure 12. Sub-clustering analysis of neurons in LRP10 OE mouse brains. A) UMAP visualization of neuronal subtypes from the sub-clustering analysis of all neurons. B) Proportions of neuronal subtypes in each experimental group. C) Multi-set intersection analysis of the sex and ApoE4 specific gene signatures in the neuron subtype from the comparisons including Female LRP10 OE E4FAD versus Female E4FAD ctrl and Male LRP10 OE E4FAD versus Male E4FAD ctrl. In each signature, the up- and down-regulated DEGs were separated for the intersection analysis. The matrix of solid and empty circles at the bottom illustrated the “presence”or “absence”of the DEG sets in each intersection. The numbers to the right of the matrix were set sizes. The colored bars on the top of the matrix represented the overlap sizes with the color intensity showing p value. [file 13024_2023_624_MOESM27_ESM.tiff]

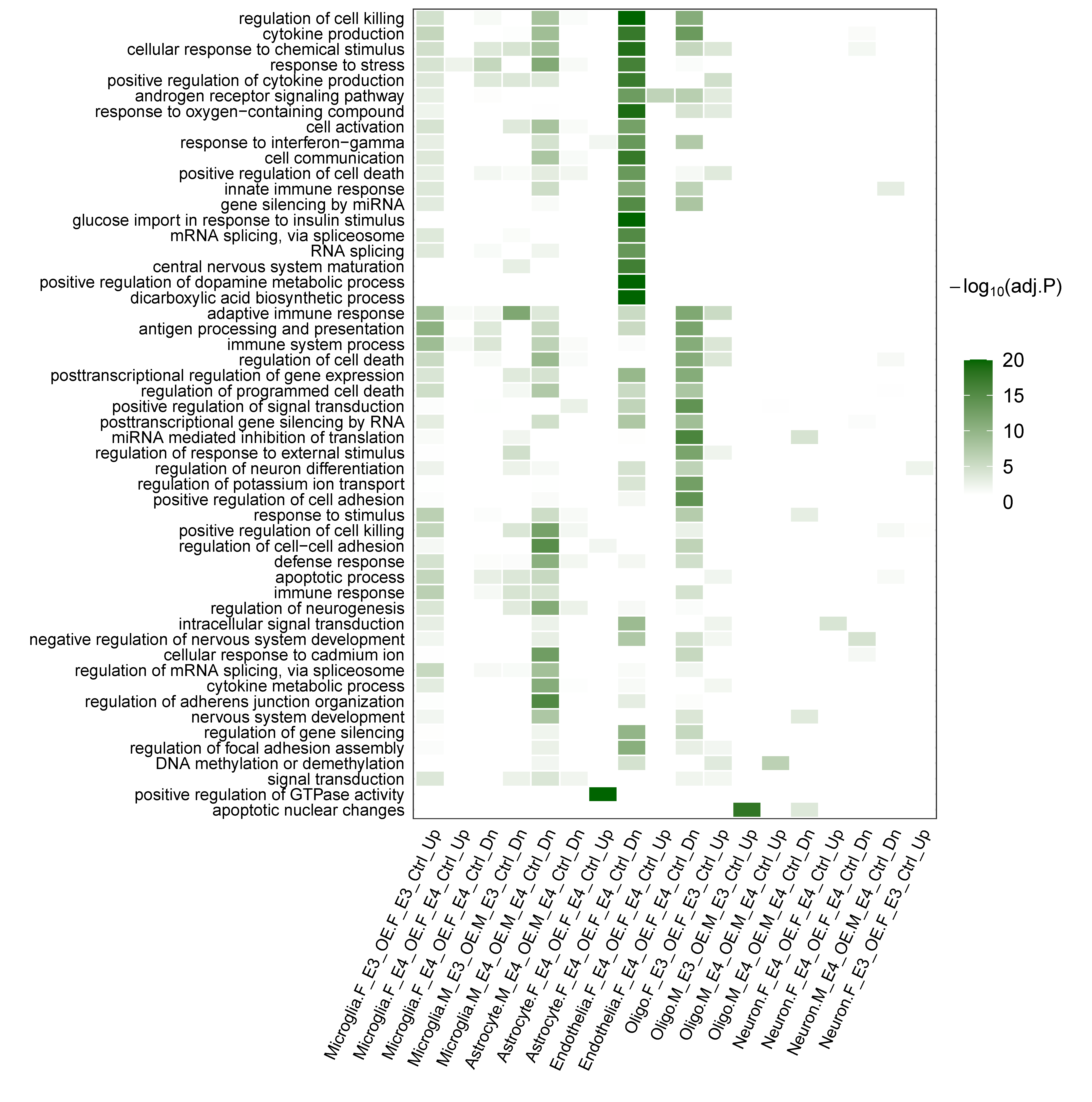

Supplement: Supplementary file 28 — Additional file 28: Supplemental Figure 13. Enrichment of functional pathways in the cell type, sex and ApoE-genotype specific gene signatures induced by LRP10 OE. [file 13024_2023_624_MOESM28_ESM.tiff]

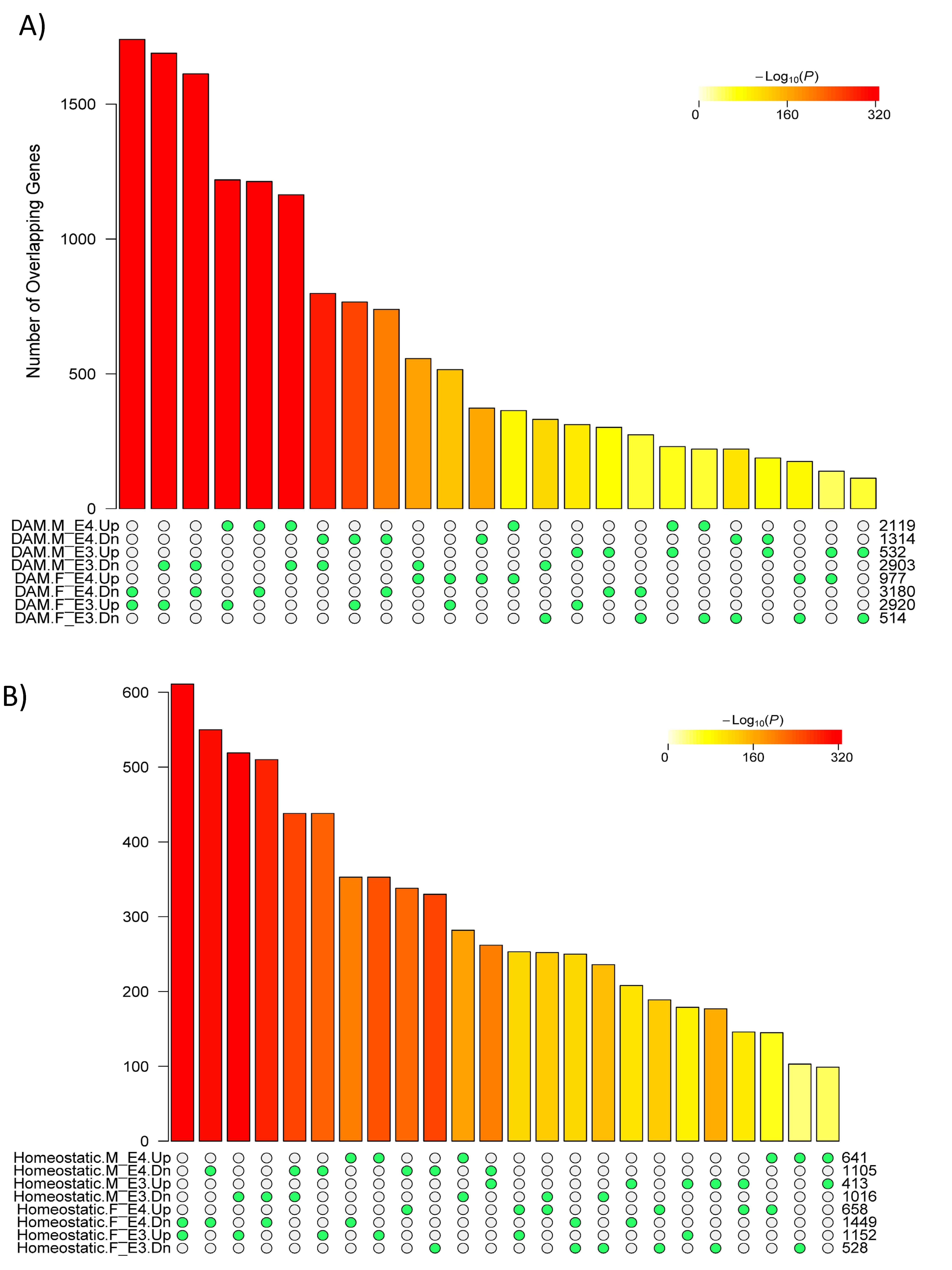

Supplement: Supplementary file 29 — Additional file 29: Supplemental Figure 14. Multi-set intersection analysis of the sex and ApoE-genotype specific gene signatures in in DAM and HAM induced by LRP10 OE. The matrix of solid and empty circles at the bottom illustrated the “presence”or “absence”of the DEG sets in each intersection. The numbers to the right of the matrix were set sizes. The colored bars on the top of the matrix represented the overlap sizes with the color intensity showing p value. [file 13024_2023_624_MOESM29_ESM.tiff]

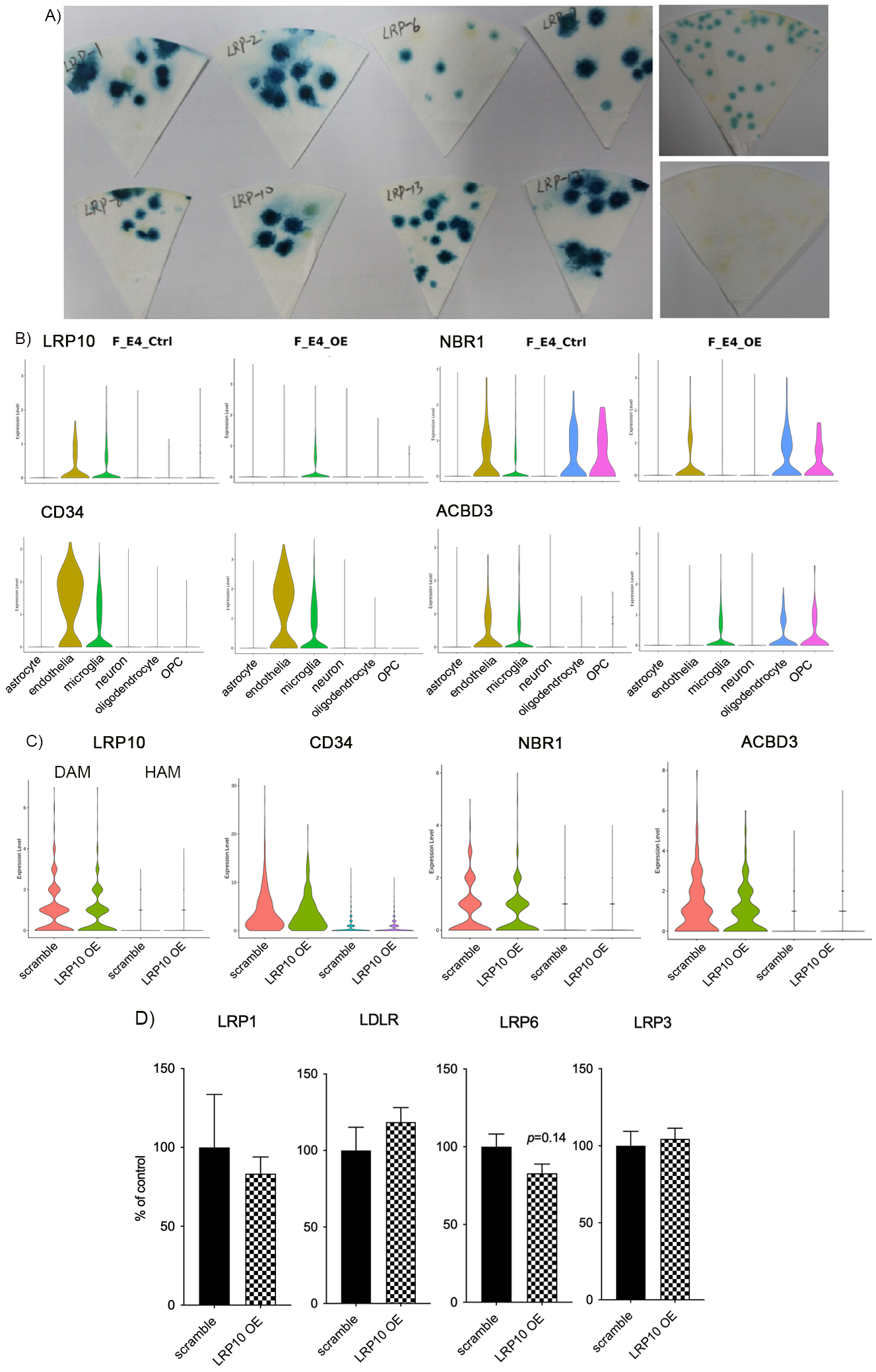

Supplement: Supplementary file 30 — Additional file 30: Supplemental Figure 15. LRP10 binding partners. A) The positive hits that were validated by β-galactosidase assays. B) The brain cell type specific expression patterns of LRP10 and its binding partners CD34, NBR1 and ACBD3 in female E4FAD mouse brains of scramble control versus LRP10 OE. C) The expression patterns of LRP10 and its binding partners CD34, NBR1 and ACBD3 in microglial subclusters such as damage-associated microglia and homeostasis-associated microglia in female E4FAD mouse brains of scramble control versus LRP10 OE. D) The levels of LRP1, LDLR, LRP6 and LRP3 protein were examined in female E4FAD mouse brains of scramble control versus LRP10 OE. N=3-9/group; *p<0.05 by unpaired T-tests with Welch’s corrections. [file 13024_2023_624_MOESM30_ESM.tif]
